# MBNL overexpression rescues cardiac phenotypes in a myotonic dystrophy type 1 heart mouse model

**DOI:** 10.1172/JCI186416

**Published:** 2025-02-11

**Authors:** Rong-Chi Hu, Yi Zhang, Larissa Nitschke, Sara J. Johnson, Ayrea E. Hurley, William R. Lagor, Zheng Xia, Thomas A. Cooper

**Affiliations:** 1 Department of Pathology and Immunology, and; 2 Department of Integrative Physiology, Baylor College of Medicine, Houston, Texas, USA.; 3 Department of Biomedical Engineering, Oregon Health and Science University, Portland, Oregon, USA.; 4 Department of Molecular and Cellular Biology, Baylor College of Medicine, Houston, Texas, USA.; 5 Center for Biomedical Data Science, Oregon Health and Science University, Portland, Oregon, USA.

**Keywords:** Genetics, Therapeutics, Arrhythmias, Cardiovascular disease, Genetic diseases

## Abstract

Myotonic dystrophy type 1 (DM1) is an autosomal dominant disease caused by a CTG repeat expansion in the dystrophia myotonica protein kinase (*DMPK*) gene. The expanded CUG repeat RNA (CUG_exp_ RNA) transcribed from the mutant allele sequesters the muscleblind-like (MBNL) family of RNA-binding proteins, causing their loss of function and disrupting regulated pre-mRNA processing. We used a DM1 heart mouse model that inducibly expresses CUG_exp_ RNA to test the contribution of MBNL loss to DM1 cardiac abnormalities and explored MBNL restoration as a potential therapy. AAV9-mediated overexpression of MBNL1 and/or MBNL2 significantly rescued DM1 cardiac phenotypes including conduction delays, contractile dysfunction, hypertrophy, and misregulated alternative splicing and gene expression. While robust, the rescue was partial compared with reduced CUG_exp_ RNA and plateaued with increased exogenous MBNL expression. These findings demonstrate that MBNL loss is a major contributor to DM1 cardiac manifestations and suggest that additional mechanisms play a role, highlighting the complex nature of DM1 pathogenesis.

## Abstract 2

This paper explored the potential of Muscleblind-like family of RNA-binding protein restoration as a potential therapy in a Myotonic Dystrophy type 1 heart mouse model.

## Introduction

Myotonic dystrophy type 1 (DM1) is an autosomal dominant multisystemic disease with a mutation prevalence of 1:2,100 that affects skeletal muscle, heart, CNS, and gastrointestinal tissues (1, 2). Over 50% of DM1-affected individuals have cardiac symptoms, predominantly conduction abnormalities and life-threatening arrhythmias, accounting for a 25% mortality rate and making this the second leading cause of death following respiratory failure (3, 4). Cardiac conduction abnormalities include prolongation of the PR (sinoatrial node to the ventricles), QRS (ventricular depolarization), and QTc (ventricular repolarization) conduction intervals (5, 6). Individuals affected by DM1 have various arrhythmias, the more common of which are sinus node dysfunction, atrial flutter, atrial fibrillation, atrioventricular block, and supraventricular tachycardia (3, 5–8). In addition, a proportion of individuals with DM1 have functional and structural abnormalities, such as left ventricular (LV) systolic dysfunction, LV dilatation, LV hypertrophy, and reduced right ventricular ejection (3, 9).

DM1 is caused by an expanded CTG repeat tract in the 3′-UTR of the dystrophia myotonica protein kinase (*DMPK*) gene (10). The mutant allele ranges from 50 to more than 4,000 repeats compared with 5–35 repeats in unaffected individuals (11, 12). Pathogenesis is caused by the RNA transcribed from the mutant *DMPK* allele containing expanded CUG repeat tracks (CUG_exp_ RNA) that accumulate in nuclear RNA foci (13, 14). The structure formed by the CUG_exp_ RNA binds and sequesters the muscleblind-like (MBNL) family of RNA binding proteins, leading to loss of function, which has been shown to be a predominant pathogenic mechanism in DM1 (12, 15).

The MBNL family consists of 3 paralogs — MBNL1, MBNL2, and MBNL3. MBNL1 and MBNL2 are ubiquitously expressed in adult tissues, with MBNL1 predominating in muscle and heart, MBNL2 being enriched in the brain, and MBNL3 being barely detectable in adult tissues (16–18). MBNL1 and MBNL2 protein levels increase during postnatal development, playing crucial roles in regulating alternative splicing and polyadenylation for hundreds of genes. This regulation is essential for the expression of adult protein isoforms (16, 19–22).

Multiple studies have shown that loss of MBNL activity reproduces physiological and molecular features of DM1 in the heart (23–26). In particular, compound loss of MBNL1 and MBNL2 exhibits physiological and molecular effects that recapitulate DM1 heart features, including cardiac arrhythmias and conduction block (23, 26). Cardiac-specific *Mbnl* double-knockout (*Mbnl1^–/–^*; *Mbnl2^cond/cond^*; *Myh6-Cre^+/–^*) results in dilated fibrotic hearts with conduction abnormalities, including prolongation of PR, QRS, and QTc intervals, as well as multiple lethal cardiac events (23). However, the extent to which increased MBNL expression rescues DM1 cardiac physiological and molecular defects induced by CUG_exp_ RNA remains to be determined.

We previously generated a bitransgenic mouse model (CUG960) of heart-specific, doxycycline-inducible (dox-inducible) expression of RNA containing 960 interrupted CUG repeats in the human *DMPK* 3′-UTR (Supplemental Figure 1A; supplemental material available online with this article; https://doi.org/10.1172/JCI186416DS1) (27). Mice with induced CUG960 (CUG960 +dox) consistently display DM1-like abnormalities including conduction abnormalities, arrhythmias, nuclear RNA foci with MBNL colocalization, and splicing defects as observed in DM1 heart tissue (5, 6, 14, 27). Importantly, the phenotypic and molecular effects are reversed after reduction of CUG_exp_ RNA levels by halting dox administration to turn off the transgene (27).

In this study, we tested the degree to which heart-specific expression of MBNL1, MBNL2, or both proteins reverses DM1-associated cardiac phenotypes and misregulated splicing in CUG960 mice. Adeno-associated virus–mediated (AAV-mediated), heart-specific expression of exogenous MBNL proteins was more than 2-fold above endogenous levels throughout the ventricles and atria of CUG960 +dox mice and primarily colocalized with CUG_exp_ RNA foci. The degree of rescue by MBNL1 and/or MBNL2 was compared side by side with the maximum level of rescue observed by turning off the transgene.

We found that both MBNL1 and MBNL2 substantially rescued the DM1 physiological and structural abnormalities induced by CUG_exp_ RNA including conduction delays, reduced cardiac contractility, and cardiac hypertrophy. RNA-Seq analysis revealed the rescue of misregulated alternative splicing and differential gene expression (DGE) events, especially those linked to disease-associated cardiac function and morphology. However, rescue of physiological, structural, and molecular disruption induced by CUG_exp_ RNA reached consistent plateaus of 50% with increased MBNL expression 2- to 3-fold above endogenous MBNL levels. The results directly support the key role that MBNL loss of function plays in DM1 cardiac pathogenesis and suggest a role for additional disease mechanisms contributing to DM1 cardiac pathogenesis.

## Results

### AAV-mediated overexpression of MBNL1 and MBNL2 is robust throughout the ventricles and atria of the CUG960 DM1 heart mouse model.

We used our established CUG960 DM1 heart mouse model (27) to determine the extent to which MBNL overexpression rescues phenotypes caused by CUG_exp_ RNA. CUG960 animals are bitransgenic for the TREDT960I transgene containing 960 interrupted CTG repeats in the context of the human *DMPK* gene driven by the tetracycline response element (TRE) promoter. A second transgene expresses reverse tetracycline transactivator (rtTA) driven by the cardiomyocyte-specific α–myosin heavy chain (α-MHC) promoter (28) (Supplemental Figure 1A). Transgene expression of CUG_exp_ RNA is induced by feeding mice dox-containing chow starting from P1, and CUG960 +dox mice recapitulate the electrophysiological and molecular DM1 cardiac features (27).

To select MBNL isoforms for expression, we first identified the MBNL1 and MBNL2 protein isoforms that predominate in adult mouse and human heart tissues based on RNA-Seq data from our laboratory (21) and the Genotype-Tissue Expression (GTEx) dataset. The predominate MBNL1 and MBNL2 splice variants in mouse and human heart tissues share 99% and 98 % amino acid sequence identities, respectively. Given the high level of identities and to assess the potential of using MBNL overexpression as a therapeutic option for DM1, we chose to use human MBNL1 and MBNL2 (Supplemental Figure 1B) to test for the rescue of cardiac defects in the CUG960 heart model.

**Figure 1:**
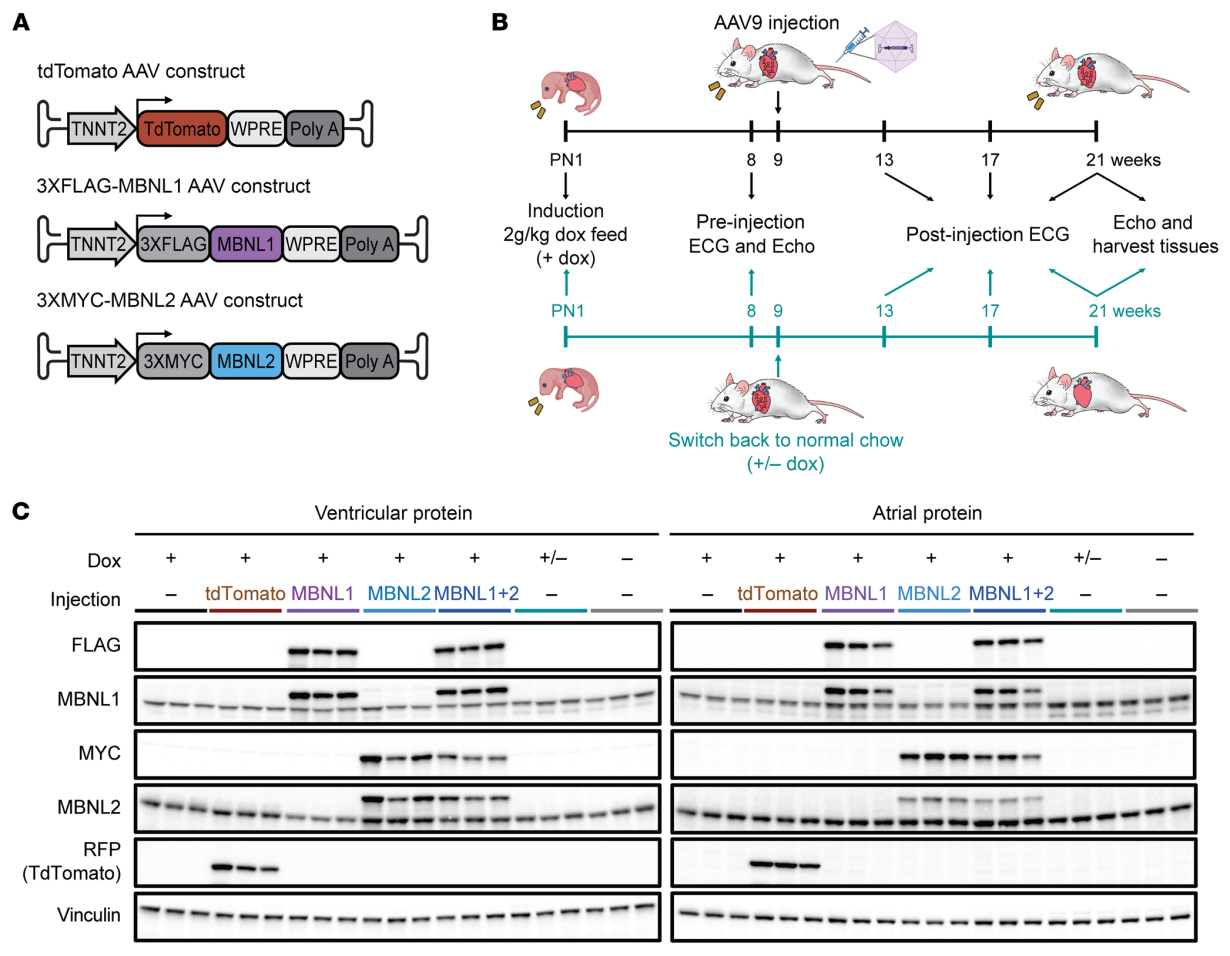
Exogenous MBNL1 and MBNL2 are overexpressed in ventricles and atria of CUG960 +dox mice by AAV9 delivery. (**A**) Diagram of the pAAV vectors used to express control tdTomato protein (pAAV-tdTomato), 3xFLAG-MBNL1, and 3xMYC-MBNL2. (**B**) Diagram of the experimental design including time points and assays. (**C**) Cardiac ventricular and atrial protein expression was evaluated by Western blotting. *n* = 3 animals per cohort.

We generated three AAV9 vectors encoding: (a) tdTomato control; (b) 3xFLAG-hMBNL1; and (c) 3xMYC-hMBNL2, each driven by the heart-specific human *TNNT2* promoter (29) (Figure 1A). CUG_exp_ RNA was induced in CUG960 +dox cohorts at P1 by feeding 2 g dox/ kg chow to nursing dams. Phenotypes of the animals were assayed at 8 weeks of age, and then AAV vectors were systemically delivered by tail vein injection into 9-week-old CUG960 +dox mice or control cohort mice using 1.69 × 10^12^ genome copies (Figure 1B). The 3 experimental cohorts expressed MBNL1, MBNL2, or MBNL1 and MBNL2 (MBNL1+MBNL2). There were 4 control cohorts: [a] CUG960 +dox and no AAV delivery; [b] CUG960 +dox injected with AAV9-tdTomato; (c) CUG960 without dox (–dox); and (d) to achieve a baseline of maximal reversal of physiological and molecular phenotypes, the fourth control cohort was CUG960 +dox mice taken off dox (+/–dox) when the other cohorts were injected with AAV9. To evaluate whether MBNL overexpression rescues cardiac conduction defects in CUG960 +dox mice, we conducted surface electrocardiography (ECG) recordings in anesthetized mice at 8 weeks of age, 1 week before AAV delivery at 9 weeks of age, and then every 4 weeks until 12 weeks after delivery (21 weeks of age) when the experiment was terminated and tissue was collected (Figure 1B).

To determine the distribution of exogenously expressed proteins in the transduced hearts, we performed immunofluorescence (IF) staining for tdTomato on frozen sagittal sections of CUG960 +dox control and AAV9-tdTomato hearts using an anti-RFP antibody. The results showed that tdTomato expression was well distributed throughout both the ventricles and atria (Supplemental Figure 1C). To quantify the expression of MBNL proteins from the AAV constructs, ventricular and atrial tissues from mice in all 7 cohorts were collected 12 weeks after delivery (21 weeks of age), and the epitope-tagged MBNL proteins were detected by Western blotting (Figure 1C). We also used anti-MBNL1 and anti-MBNL2 antibodies that recognized both mouse and human homologs to compare exogenous and endogenous proteins on the same Western blots (Figure 1C). Compared with endogenous MBNL1 levels in the CUG960 +dox control cohort, total MBNL1 expression levels (endogenous + exogenous MBNL1) were increased 3.7-fold in ventricles and 2.9-fold in atria for the MBNL1 cohort, and 3.9-fold in ventricle and 2.7-fold in atria for the MBNL1+MBNL2 cohort (Figure 1C and Supplemental Figure 1D). Total MBNL2 expression was increased 3.4-fold in ventricles and 2.1-fold in atria of the MBNL2 cohort compared with MBNL2 levels in CUG960 +dox control, and 2.4-fold in ventricles and 1.7-fold in atria in the MBNL1+MBNL2 cohort (Figure 1C and Supplemental Figure 1D). Overall, we confirmed that expression of the AAV9-delivered, epitope-tagged MBNL proteins was robust and well distributed in both the ventricles and atria.

### MBNL overexpression rescues the conduction defects caused by CUG_exp_ RNA.

**Figure 2:**
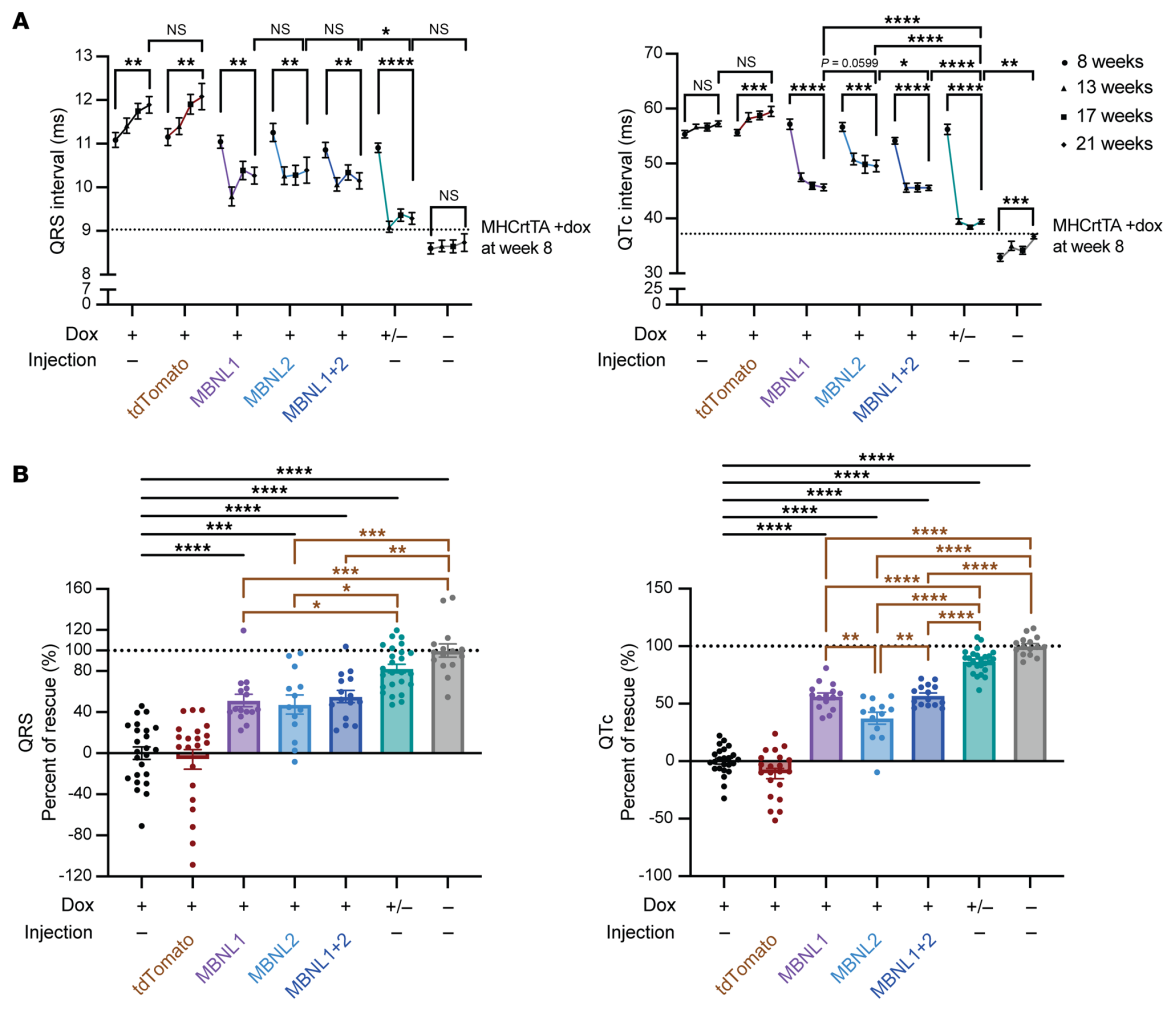
Cardiac conduction intervals were rescued by overexpression of MBNL1 and/or MBNL2. (**A**) QRS (left) and QTc (right) intervals were determined by surface ECG recordings in anesthetized CUG960 mice in response to dox induction with different treatments. On/off dox (+/–) animals were taken off dox at the time of AAV9 delivery and served as a control for complete recovery. Data on QRS and QTc intervals from MHCrtTA +dox control animals at 8 weeks of age are indicated by a dashed line (28). *n* ≥13 per cohort. Data represent the mean ± SEM and were analyzed using 2-way ANOVA followed by Tukey’s multiple-comparison test. (**B**) Percent rescue of QRS (left) and QTc (right) intervals was calculated using the average ECG parameters from the last time point (21 weeks of age). *n* ≥13 per cohort. Black lines represent the significant differences of corresponding groups compared with +dox or tdTomato controls; brown lines represent the significant differences between the corresponding groups and +/–dox and –dox controls. Data represent the mean ± SEM. *P < 0.05, **P < 0.01, ***P < 0.001, and ****P < 0.0001, by 1-way ANOVA followed by Tukey’s multiple-comparison test.

As shown in our previous study (27), CUG960 +dox mice showed prolongation of the QRS and QTc intervals in comparison with CUG960 –dox controls, whereas CUG960 +/–dox mice showed rescue of these parameters nearly to the levels seen in CUG960 –dox controls (Figure 2A). MBNL1 and/or MBNL2 overexpression showed a significant reduction of prolonged QRS and QTc intervals in all 3 experimental cohorts (Figure 2A). To determine the levels of rescue in each cohort, we set the range of the average ECG parameters from the last time point (12 weeks after AAV delivery, 21 weeks of age) for the CUG960 +dox cohort as 0% rescue and that of the CUG960 –dox cohort as 100% rescue. Rescue of QRS intervals in response to overexpression of MBNL1, MBNL2, and MBNL1+MBNL2 was 52.4%, 35%, and 55.2%, respectively. Rescue of QTc prolongation was 56.4%, 31.7%, and 56.9% upon overexpression of MBNL1, MBNL2, and MBNL1+MBNL2, respectively (Figure 2B). Interestingly, while mice in the MBNL1 and MBNL2 cohorts showed improved ECG parameters, those in the MBNL1+MBNL2 cohort did not exhibit an additive effect on the level of rescue for either QRS or QTc intervals, indicating that we reached the maximum level of rescue that can be achieved with MBNL overexpression. In contrast to the partial rescue observed due to MBNL overexpression, both QRS and QTc intervals were almost completely rescued by shutoff of the CUG_exp_ RNA in CUG960 +/–dox mice (Figure 2B). These results suggest that either overexpression of exogenous MBNL did not completely restore endogenous MBNL activity, or that exogenous MBNL restored endogenous MBNL activity, and mechanisms, in addition to MBNL loss of function, play a role in the cardiac phenotypes induced by CUG_exp_ RNA in the CUG960 mouse model (see Discussion).

In an independent set of experiments, we investigated the duration of MBNL-mediated rescue after delivery. We systemically delivered 10^12^ genome copies of an AAV9 vector for heart-specific expression of both FLAG-tagged MBNL1 and MYC-tagged MBNL2 into 8-week-old CUG960 +dox mice (induced at P1) (Supplemental Figure 2A). CUG960 +dox mice given AAV9-mCherry served as a control along with CUG960 +dox–only and CUG960 +/–dox control cohorts. Predelivery ECG measurements were recorded in mice at 7 weeks of age, and then post-delivery measurements were recorded monthly for 5 months (28 weeks of age) (Supplemental Figure 2B). The expression of FLAG-tagged MBNL1 and MYC-tagged MBNL2 as well as the control mCherry protein was detected by Western blotting (Supplemental Figure 2C). We found that overexpression of MBNL1 and MBNL2 rescued the prolonged QRS and QTc intervals in CUG960 +dox mice at the first month after delivery (12 weeks of age); moreover, the degree of QRS and QTc interval rescue was maintained for the entirety of the 5-month experiment (Supplemental Figure 2D). These results independently demonstrated that the conduction deficits caused by CUG_exp_ RNA could be partially rescued by cardiac-specific overexpression of MBNL proteins and that rescued parameters were maintained for at least 5 months after delivery.

### MBNL overexpression rescues the cardiac morphology and contractile defects caused by CUG_exp_ RNA.

**Figure 3:**
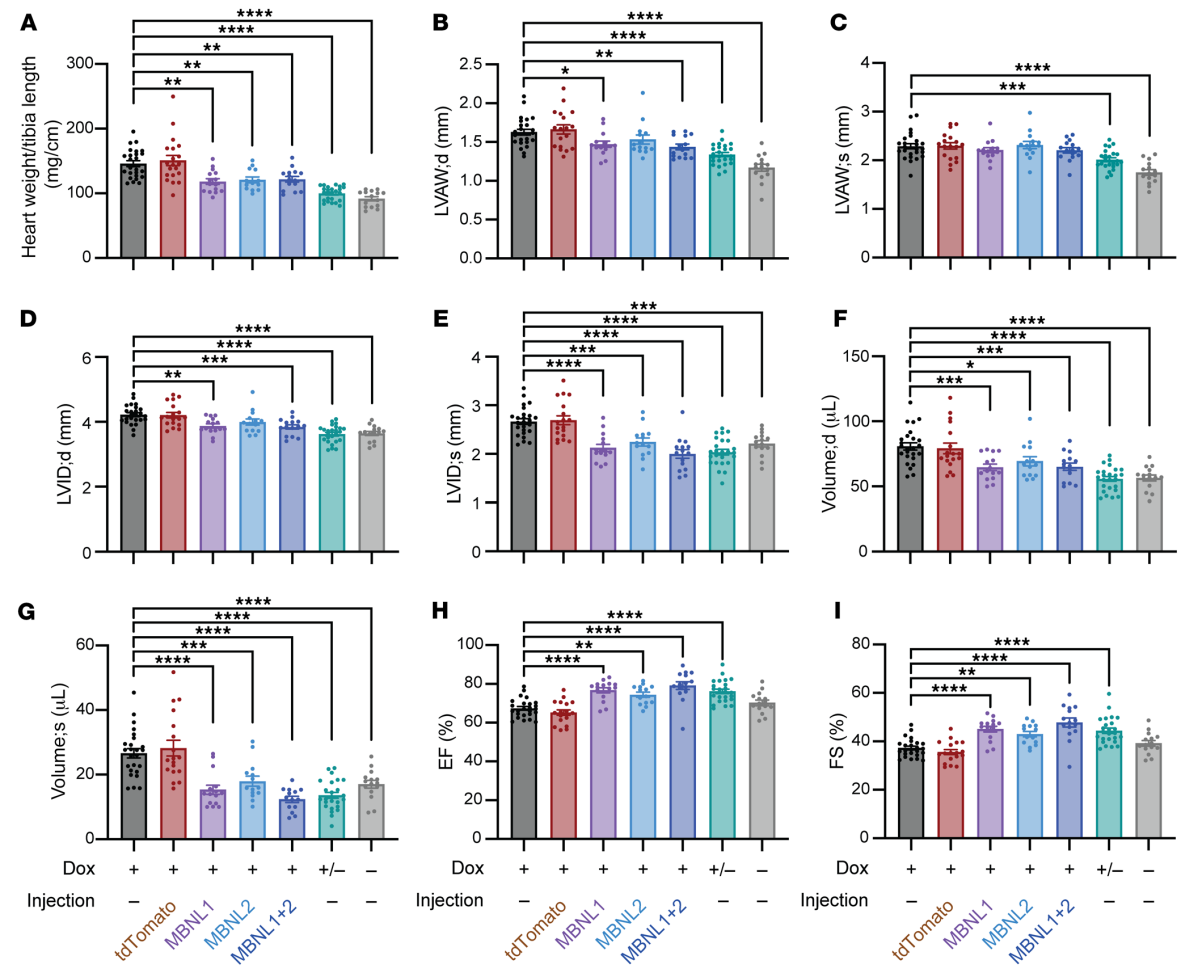
Heart weight and echocardiographic parameters induced by CUG_exp_ RNA show significant or trending rescue with MBNL1 and/or MBNL2 overexpression. (**A**) Heart weight was normalized to tibia length. (**B** and **C**) Left ventricle anterior wall (LVAW) thickness, (**D** and **E**) left ventricle internal diameter (LVID), (**F** and **G**) LV volume, (**H**) ejection fraction (EF), and (**I**) fractional shortening (FS) were determined by M-mode Echo. *n* ≥13 per cohort. All animals were analyzed at the 21-week time point. Data represent the mean ± SEM and were analyzed by ordinary 1-way ANOVA. *P < 0.05, **P < 0.01, ***P < 0.001, and ****P < 0.0001. d, end of diastole; s, end of systole.

To determine the degree to which MBNL overexpression rescued the cardiac structural changes induced by CUG_exp_ RNA, we measured the heart weight/tibia length ratios. In comparison with CUG960 –dox mice, the CUG960 +dox mice had significantly increased heart weight/tibia length ratios (59.3% increase) (Figure 3A). To measure changes in cardiac contractile function and structure, we performed echocardiography (Echo) in the short-axis view. M-mode images showed significant increases in LV anterior wall (LVAW) thickness, LV internal diameter (LVID) in both end of systole and diastole, and LV mass in the CUG960 +dox mice (Figure 3, B–E, and Supplemental Figure 3A). In addition, systolic and diastolic end volumes were both significantly increased, while the ejection fraction and fractional shortening were both significantly reduced in the CUG960 +dox mice (Figure 3, F–I). These data indicate that CUG960 +dox mice had enlarged hearts with muscular hypertrophy and compromised LV contractility, similar to the abnormal cardiac features observed in DM1 (23, 24, 26). All these parameters were reversed after shutting off CUG_exp_ RNA expression by withdrawing dox chow. Importantly, the hypertrophy and reduced contractility induced by CUG_exp_ RNA were significantly rescued by overexpression of MBNL1 and/or MBNL2, approaching the values seen in CUG960 –dox and +/–dox mice (Figure 3 and Supplemental Figure 3).

### Exogenous MBNL1 and MBNL2 are colocalized with CUG_exp_ RNA foci.

**Figure 4:**
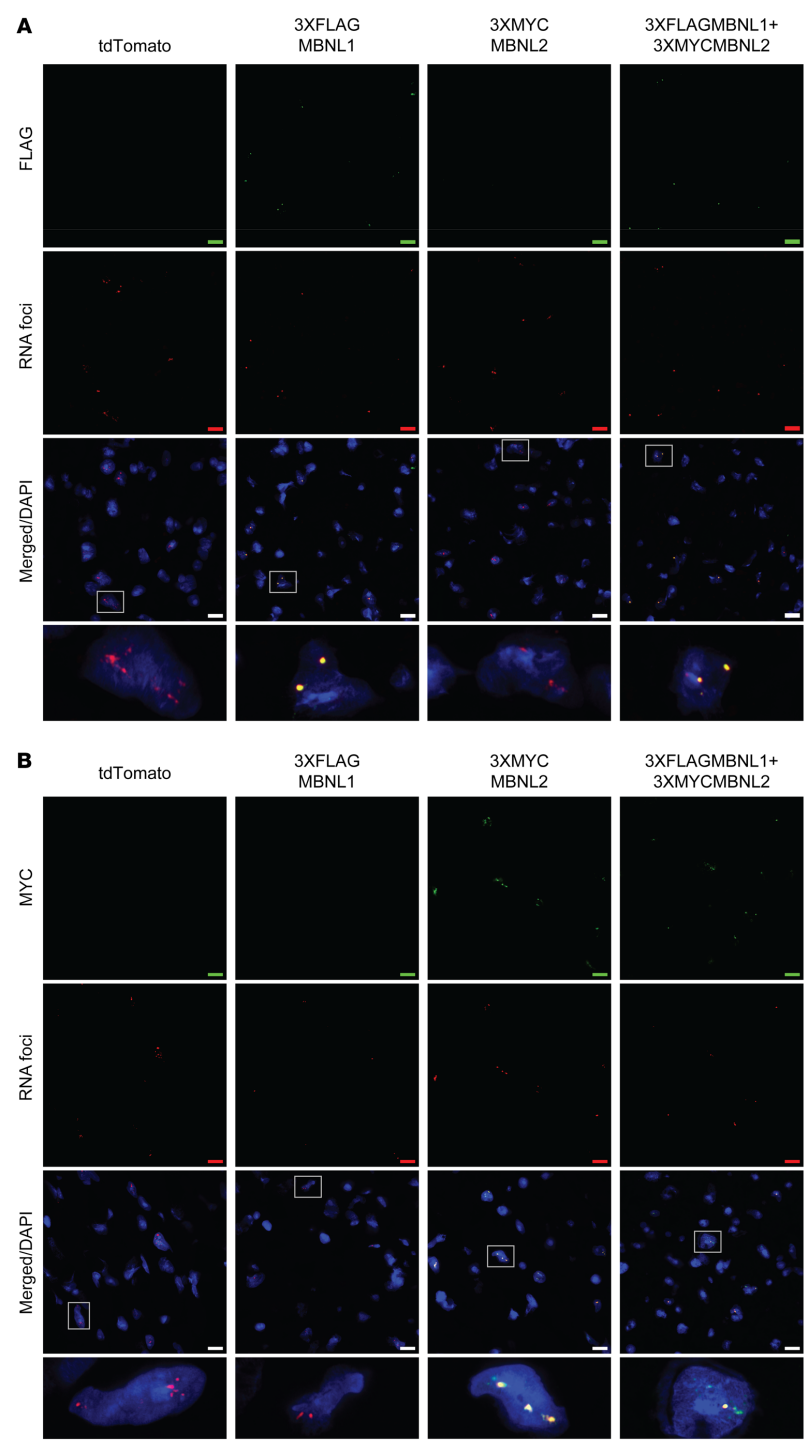
CUG960 +dox mice with MBNL overexpression show colocalization of epitope-tagged MBNL1 and MBNL2 with nuclear CUG_exp_ RNA foci. RNA FISH using a (CAG)_5_ locked nucleic acid probe targeting CUG_exp_ RNA combined with IF for (**A**) FLAG tag and (**B**) MYC tag in the ventricles of CUG960 +dox mice. The experiments were conducted on 3 animals in each group, and representative images are shown. Scale bars: 10 μm.

One of the key hallmarks of DM1 is the formation of nuclear foci and sequestration of MBNL proteins by pathogenic CUG_exp_ RNA, which is observed in DM1 cardiac tissues, as well as in the CUG960 mouse model (14, 27). To determine the distribution of epitope-tagged MBNL proteins relative to CUG_exp_ RNA foci, we performed FISH using Cy5-labeled (CAG)_5_ locked nucleic acid probes targeting CUG_exp_ RNA, combined with IF for FLAG, MYC, MBNL1, or MBNL2. Both FLAG (MBNL1) and MYC (MBNL2) signals showed pronounced colocalization with the nuclear RNA foci in both the ventricles and atria (Figure 4, A and B). Specifically, FLAG signals (MBNL1) were observed in mice with MBNL1 and MBNL1+MBNL2 overexpression and MYC signals (MBNL2) were identified in MBNL2 and MBNL1+MBNL2 cohorts (Figure 4, A and B). IF staining for MBNL proteins showed colocalization of MBNL1 and MBNL2 proteins with nuclear RNA foci in all animals with dox chow, but we observed no foci or MBNL protein sequestration in CUG960 –dox control (Supplemental Figure 4).

Surprisingly, we observed that the mean foci count per nucleus significantly decreased in ventricles of mice in the MBNL1 and MBNL1+MBNL2 cohorts compared with tdTomato control mice, with no change in foci signal intensity of the remaining foci (Supplemental Figure 5, A and B). To determine whether AAV9-delivered overexpression of MBNL proteins affects the expression of CUG_exp_ RNA, we measured relative expression levels of CUG_exp_ RNA by quantitative reverse transcription PCR (RT-qPCR) analysis in the atria and ventricles of mice in each cohort. In comparison with CUG960 –dox control mice, CUG960 +dox mice showed a robust induction of transgene expression, whereas this expression was extinguished in response to dox withdrawal in CUG960 +/–dox mice as shown previously (27) (Supplemental Figure 5C). However, we observed no significant changes in expression of the transgene in the ventricles of mice with MBNL1 and/or MBNL2 overexpression compared with expression in the ventricles of CUG960 +dox mice (Supplemental Figure 5C). MBNL1 overexpression yielded variable results, with no change observed with some assays or, less often, a trend toward slightly reduced expression, as shown in Supplemental Figure 5C.

Taken together, the data showed robust expression of FLAG and MYC-tagged MBNL proteins colocalizing with foci. Moreover, overexpression of the epitope-tagged MBNL proteins reduced the foci count per nucleus, with no change in foci intensity or overall expression of CUG_exp_ RNA from the transgene.

### MBNL overexpression rescues disrupted splicing events related to the cardiac morphology and function caused by CUG_exp_ RNA.

A downstream consequence of CUG_exp_ RNA expression and MBNL loss of function is the disruption of developmentally regulated alternative splicing with target transcripts showing reversion to fetal isoforms. To determine the degree of transcriptome rescue in response to MBNL overexpression, we performed RNA-Seq analysis on poly(A)-selected RNA from ventricles and atria of three 21-week-old animals from each cohort (Supplemental Table 1). We acquired an average of 109 million paired-end, 150 bp reads with greater than 58% of reads uniquely mapping to the mouse genome. Correlation plots for ventricular (Supplemental Figure 6) and atrial (Supplemental Figure 7) gene expression showed a strong correlation between the 3 biological replicates. Principal component analysis (PCA) revealed strong separation between ventricle and atrium transcriptomes on PC1 (Supplemental Figure 8). On PC2 of both ventricle and atrium transcriptomes, CUG960 +/–dox clustered close to the CUG960 –dox cohort, separate from the CUG960 +dox cohort. Moreover, all 3 MBNL cohorts clustered separately between the CUG960 +dox and –dox cohorts (Supplemental Figure 8). Overall, the RNA-Seq results showed high levels of reproducibility between the biological samples.

**Figure 5:**
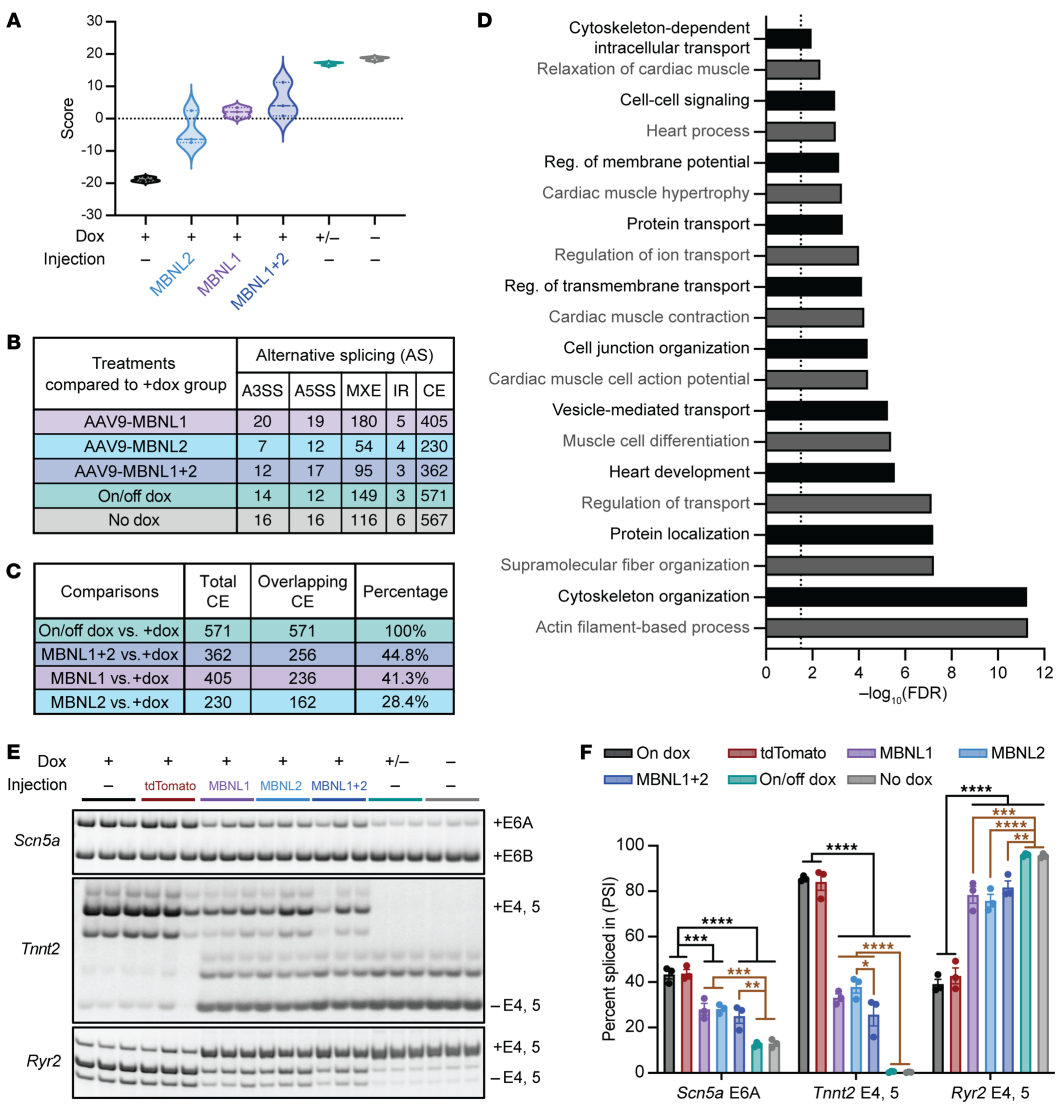
MBNL overexpression partially rescues disrupted ventricular splicing events related to cardiac morphology and function. (**A**) Alternative splicing signature scores for ventricle samples. The center line indicates the median value, while the upper and lower dashed lines indicate the first and third quartiles. (**B**) Number and types of alternative splicing events observed in ventricles. IR, intron retention. (**C**) Percentage of overlapping CE splicing events in each MBNL-overexpressing cohort using the comparison between CUG960 +/–dox and +dox cohorts as a reference. (**D**) Genes showing differential alternative splicing changes in comparisons of CUG960 –dox mice with +dox mice, as well as in comparisons of all MBNL cohorts to CUG960 +dox cohort were evaluated for enrichment of GO functional terms using the ShinyGO platform. The dashed vertical line: –log_10_(FDR) = 1.5 (**E**) Representative polyacrylamide gels showing alternative splicing changes for the candidate genes *Scn5A*, *Tnnt2*, and *Ryr2* in ventricles of CUG960 +dox mice that underwent different treatments. (**F**) Quantification of the PSI for each splicing event. *n = 3* animals per cohort. Black lines represent the significant differences in the corresponding groups compared with +dox or tdTomato controls; brown lines represent the significant differences between the corresponding groups and +/–dox and –dox controls. E, exon. Data represent the mean ± SEM. *P < 0.05, **P < 0.01, ***P < 0.001, and ****P < 0.0001, by 2-way ANOVA with Tukey’s multiple-comparison test.

To visualize the level of rescue in transcriptome-wide misregulated splicing events between cohorts, we developed a signature scoring system that quantified the degree of splicing pattern restoration by integrating all significant alternative splicing events into a comprehensive metric. We used comparisons of CUG960 +dox and CUG960 –dox as the extremes to define a disease-related signature, in which CUG960 –dox (no CUG_exp_ RNA) samples showed high positive scores and CUG960 +dox samples showed low negative scores, and used this standard to compare the degree of rescue for all cohorts in ventricles (Figure 5A) and atria (Supplemental Figure 9A) (see Methods for details). A higher score indicated a better rescue and more similarity to the unaffected splicing pattern in the CUG960 –dox samples, whereas scores that remained low suggested incomplete rescue. The alternative splicing score for +/–dox was close to the –dox control score, consistent with the expected high level of rescue upon shutoff of CUG_exp_ RNA. All 3 MBNL cohorts had similar scores at the middle of the scoring system, with MBNL1 and MBNL1+MBNL2 cohorts showing stronger rescue compared with the MBNL2-alone cohort in both ventricle and atrium samples (Figure 5A and Supplemental Figure 9A).

We identified differential alternative splicing patterns by comparing each cohort with CUG960 +dox, with the cutoff of an FDR below 0.05 and a ΔPSI³ of 15% (Figure 5B and Supplemental Figure 9B). The comparison of CUG960 +/–dox cohort with the CUG960 +dox cohort identified a total of 749 ventricular and 694 atrial differentially spliced events, similar to the results from the comparison of the CUG960 –dox cohort with the CUG960 +dox cohort and indicative of a high level of rescue upon shutoff of CUG_exp_ RNA (Figure 5B and Supplemental Figure 9B). Given that changes in cassette exon (CE) splicing account for approximately 80% of these altered splicing events, the comparison of CUG960 +/–dox to +dox CE events was used as a reference for the maximum level of rescue to determine the levels of rescue in MBNL-overexpressing cohorts. We observed that there were 571 splicing events affected by CUG_exp_ expression and were rescued once CUG_exp_ RNA was removed from ventricles in the CUG960 +/–dox cohort (Figure 5C). Within these 571 splicing events, there were 256 events that were significantly rescued by MBNL1+MBNL2 overexpression, which indicates that 44.8% of these splicing events were rescued with MBNL1+MBNL2 expression in ventricles (Figure 5C). We observed partial rescue in other MBNL cohorts, and the percentages of corresponding splicing events that met the cutoff for rescue due to exogenous MBNL expression were, in ventricles and atria, respectively, MBNL1+MBNL2 (44.8% and 31.8%), followed by MBNL1 (41.3% and 24.5%) and MBNL2 (28.4% and 10.4%) (Figure 5C and Supplemental Figure 9C).

Next, the alternative splicing events that were rescued by CUG_exp_ RNA shutoff and expression of single or combined MBNL paralogs were used for gene ontology (GO) analysis. The top 20 biological process pathways that were enriched through GO analysis of genes undergoing alternative splicing changes were similar in the ventricles and atria (Figure 5D and Supplemental Figure 9D). The predominant categories showed alternative splicing events affecting genes involved in the regulation of cardiac conduction, contraction, and morphology.

We performed RT-PCR analysis to validate alternative splicing events with relevance to altered cardiac physiology in CUG960 +dox mice identified by RNA-Seq (Figure 5, E and F, Supplemental Figure 9, E and F, and Supplemental Figures 10 and 11). In comparison with CUG960 –dox control mice, CUG960 +dox mice showed strong disruption of splicing events in both the ventricles and atria. These splicing disruptions include genes that control cardiomyocyte action potential (*Scn5a*, *Kcnip2*, and *Kcnd3*) and calcium handling (*Ryr2* and *Camk2d*), which have been previously shown to switch to fetal splicing isoforms upon the induction of the CUG_exp_ repeats (27) (Figure 5, E and F, Supplemental Figure 9, E and F, Supplemental Figure 10, A–C, and Supplemental Figure 11, A–C). Moreover, known MBNL targets, including *Sorbs1*, *Cacna1s*, and *Tmem63b*, were mis-spliced and shifted toward fetal isoforms. Similar mis-spliced patterns were found in genes related to cardiomyopathy and long-QT syndrome/arrhythmia, including *Plekhm2*, *Dnm1l*, and *Golga2* (23, 26) (Supplemental Figure 10, D–I, and Supplemental Figure 11, D–H). With MBNL1 and/or MBNL2 overexpression, these splicing defects were significantly but partially rescued compared with the maximal level of rescue achieved by turning off transgene expression (Figure 5, E and F, Supplemental Figure 9, E and F, and Supplemental Figures 10 and 11). These data indicate that exogenous MBNL expression partially rescued the PSI levels of mis-spliced genes related to cardiac function and morphology in CUG960 +dox mice and are consistent with the degree to which cardiac physiology was rescued.

To determine whether higher expression of exogenous MBNL results in greater splicing rescue, we looked at the relationship between the levels of MBNL expression and splicing rescue in the 5 ventricle samples from the MBNL cohort in Supplemental Figure 2C. We quantified exogenous MBNL protein levels in ventricle samples and found that exogenous MBNL1 expression in samples 1, 2, and 5 were increased 3.6-, 10.8- and 7-fold, respectively, compared with endogenous MBNL1 expression levels (Supplemental Figure 12A; samples 1, 2, and 5 are indicated in green, pink, and orange, respectively). We tested 4 splicing events in these heart samples. While different splicing events show different levels of rescue consistent with different sensitivities to MBNL1, we observed no substantial differences in rescue for each splicing event with the 7.0- and 10.8-fold (orange and pink samples) increases of MBNL1 expression compared with the 3.6-fold (green) increase of MBNL1 expression (Supplemental Figure 12B). Since exogenous MBNL2 expression was low (Supplemental Figure 12A), the contribution MBNL2 was minor. We conclude that we had reached the maximum rescue potential by MBNL overexpression in our DM1 CUG960 heart mouse model.

### MBNL overexpression rescues the gene expression changes caused by CUG_exp_ RNA.

**Figure 6:**
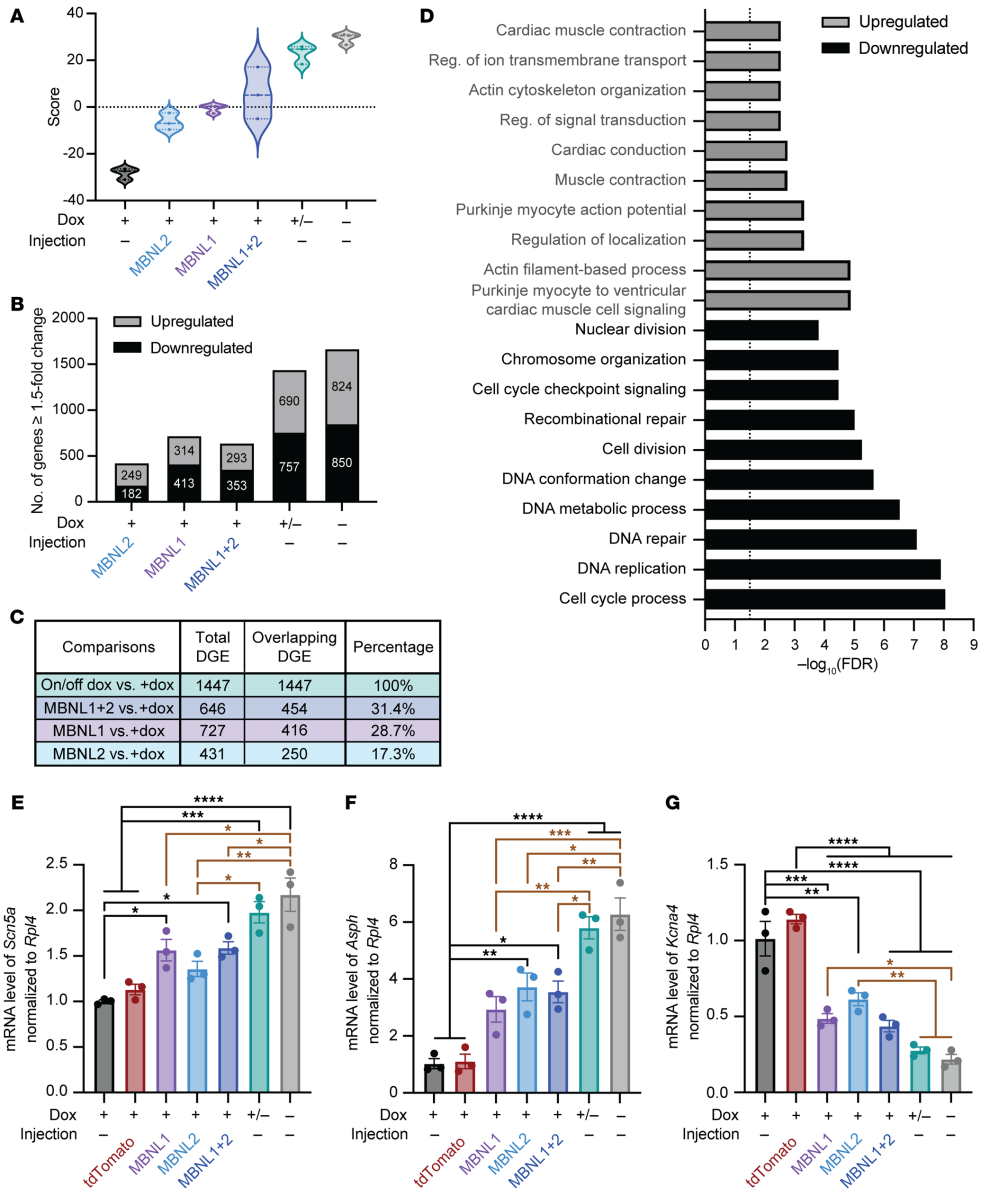
MBNL1 and/or MBNL2 overexpression results in partial rescue for widespread DGE in ventricles of CUG960 +dox mice. (**A**) Gene signature scores for ventricular samples. The center line represents the median value, while the upper and lower dashed lines indicate the first and third quartiles. (**B**) Differentially expressed genes displaying at least a 1.5-fold change (adjusted *P* = 0.05) as compared with expression in CUG960 +dox controls. (**C**) Percentages of overlapping DGE in each MBNL-overexpressing cohort using the comparison between CUG960 +/-dox and +dox cohorts as a reference. (**D**) GO functional terms for genes showing DGE changes in the ventricles of mice in all cohorts. Dashed line: –log_10_(FDR) = 1.5. (**E**–**G**) RT-qPCR–based validation of candidate genes showing gene expression changes in ventricles of mice in each cohort. *Rpl4* was used as an internal control for normalization. *n* = 3 animals per cohort. Black lines indicate the significant differences in corresponding groups compared with +dox or tdTomato controls; brown lines indicate the significant differences between the corresponding groups and +/–dox and –dox controls. Data represent the mean ± SEM. *P < 0.05, **P < 0.01, ***P < 0.001, and ****P < 0.0001, by ordinary 1-way ANOVA followed by Tukey’s multiple-comparison test. Reg., regulation.

Our previous transcriptome analysis of CUG960 +dox versus control heart tissues identified DGE changes in genes that are associated with inherited cardiac conduction diseases (27). To explore transcriptome-wide rescues of gene expression, we analyzed the RNA-Seq data for DGE with a cutoff of an adjusted *P* value of less than 0.05 and an absolute fold change of 1.5 or higher. Similar to alternative splicing, we generated a signature score system to visualize the level of rescue of global gene expression changes between cohorts. We observed a pattern similar to that seen with alternative splicing in both ventricular and atrial samples. CUG960 +/–dox samples clustered with the CUG960 –dox control, and all 3 MBNL cohorts had similar scores for partial rescue, with the MBNL2 cohort showing slightly less rescue than the MBNL1 and MBNL1+MBNL2 cohorts (Figure 6A, ventricle and Supplemental Figure 13A, atria).

Next, genes with differential expression between each cohort and the CUG960 +dox cohort were categorized into upregulated and downregulated genes (Figure 6B and Supplemental Figure 13B). The comparison of CUG960 +/–dox with CUG960 +dox mice (1,447 ventricular and 871 atrial differentially expressed genes), representing the maximal level of rescue, was again used as a reference to determine the levels of rescue in MBNL-overexpressing cohorts. The percentages of genes that passed the cutoff for rescue with exogenous MBNL expression were, in ventricles and atria, respectively, as follows: MBNL1+MBNL2 (31.4% and 7.9%), followed by MBNL1 (28.7% and 5.4%) and MBNL2 (17.3% and 0.6%) (Figure 6C and Supplemental Figure 13C).

GO analysis of ventricular DGE rescued by MBNL expression showed enrichment for various biological processes (Figure 6D). Genes related to the regulation of cardiac action potential and association with cardiac disease were chosen for validation (Supplemental Table 2). *Scn5A* levels in ventricles, as well as *Asph* and *Ryr2* levels in both ventricles and atria were decreased in the CUG960 +dox cohort compared with the –dox mice and were fully rescued by turning off transgene expression in the CUG960 +/–dox mice, whereas the rescue levels were approximately 40%–60% compared with rescue levels in +/–dox mice with overexpression of MBNL (Figure 6, E and F, Supplemental Figure 13, D–F, and Supplemental Figure 14A). *Gja5* and *Hcn4*, which have been shown to be misregulated in atria of CUG960 +dox mice (27), were not completely rescued after shutting off transgene expression in CUG960 +/–dox mice and were not rescued in all MBNL cohorts (Supplemental Figure 13, G and H). Misregulation of *Cdnf* upon CUG_exp_ RNA expression, the top hit in the upregulation category of DGE in ventricular samples, was rescued to CUG960 –dox control levels after the CUG_exp_ RNA was turned off or following overexpression of MBNL1 or MBNL1+MBNL2 proteins (Supplemental Figure 14B). In addition, upregulation of *Kcna4* and downregulation of *Ckmt2*, genes linked to dilated cardiomyopathy and arrhythmia, have been observed in cardiac-specific MBNL1 and MBNL2 double-knockout mice (23) and were also misregulated in CUG960 +dox mice (Figure 6G, Supplemental Figure 13, I and J, and Supplemental Figure 14C). MBNL overexpression in CUG960 +dox mice significantly rescued the misregulation of *Kcna4* and *Ckmt2* mRNA toward the levels detected in +/–dox and –dox control in ventricles (Figure 6G and Supplemental Figure 14C). Overall, genes that were misregulated due to CUG_exp_ repeat expression differed with regard to partial or nearly complete rescue upon overexpression of MBNL proteins.

## Discussion

While more than half of the individuals with DM1 have cardiac manifestations (3, 4), the mechanisms that contribute to cardiac disease features are not well understood. MBNL loss of function phenocopies the cardiac DM1 features in mice, indicating that sequestration and loss of MBNL function play an important role in DM1 pathogenesis (23–26, 30). In this study, we tested the degree to which the cardiac defects induced by expression of CUG_exp_ RNA can be rescued by overexpression of exogenous MBNL proteins in a DM1 cardiac mouse model. AAV9-mediated overexpression of either MBNL1 or MBNL2 alone or dual MBNL1+MBNL2 all significantly, yet partially, reversed the prolongation of cardiac conduction intervals and structural and functional cardiac abnormalities, as well as misregulated alternative splicing and the global gene expression changes induced by CUG_exp_ RNA.

Overexpression of MBNL rescued only 50% of the DM1 physiological and molecular features induced by CUG_exp_ RNA compared with the maximal level of rescue achieved with removal of CUG_exp_ RNA in the CUG960 +/–dox cohort tested in parallel. There are several possible reasons for the partial rescue. A contributing factor could be the likelihood that AAV9 does not transduce and express protein in all cardiomyocytes equally, leading to variable rescue between cardiomyocytes. Another concern was the possibility of having insufficient expression of MBNL proteins due to technical limitations of AAV delivery. To address these concerns, we confirmed that (a) AAV9-delivered tdTomato proteins were well distributed throughout ventricles and atria; (b) combined FISH for CUG_exp_ RNA and IF for epitope tags showed that the majority of RNA foci costained for exogenous MBNL proteins, demonstrating coexpression; and (c) total MBNL1 and MBNL2 protein levels averaged 2- to 3-fold above endogenous levels in MBNL overexpression cohorts in both the ventricles and atria. Although total MBNL protein levels in mice with MBNL1+MBNL2 overexpression were approximately 2 times higher than those in mice with single MBNL1 or MBNL2 overexpression, the MBNL1+MBNL2 cohort showed rescue levels similar to those seen in the single expression cohorts, indicating that this dual overexpression did not provide an additive effect. In addition, heart tissue from mice expressing a range of MBNL1 from 3.6- to 10.8-fold compared with endogenous MBNL1 expression in Supplemental Figure 2C showed only incremental differences in splicing rescue (Supplemental Figure 12). The data indicate that we reached the maximum level of rescue that could be achieved by exogenous MBNL protein expression in the presence of CUG_exp_ RNA.

Another possibility is that exogenously delivered protein cannot completely fulfill the activities of endogenous proteins. While we chose the predominant MBNL isoform in the heart for our overexpression experiments and showed that exogenous MBNL proteins were localized to the nucleus in RNA foci, other MBNL isoforms with potentially different activities or localization might be required to completely restore MBNL functionality.

MBNL1 overexpression in skeletal muscle of HSA^LR^ mice was shown to result in partial rescue for splicing events, and the level of rescue varied depending on individual mis-spliced events (31, 32). Although there is a question of how much of the failure to fully rescue is due to the challenge of providing full activity using exogenous MBNL, our results showing partial rescues raise an intriguing possibility that exogenous MBNL efficiently rescued the pathogenic component caused by MBNL loss of function and that what was not rescued were other mechanisms for DM1 cardiac pathogenesis induced by CUG_exp_ RNA. In addition to MBNL proteins, other RNA-binding proteins, including CUGBP and Elav-like family member 1 (CELF1), HNRNPA1, and the double-stranded RNA-binding protein Staufen 1 (STAU1), have been proposed to play a role in DM1 disease onset (12, 15, 33–37). Additional effects of the CUG_exp_ RNA such as repeat-associated, non-AUG (RAN) translation could contribute to cardiac pathogenesis (12, 38). These additional factors may contribute to the observed cardiac pathology.

Interestingly, we found that overexpression of MBNL proteins in CUG960 +dox mice led to a reduction in the number of CUG_exp_ RNA foci per nucleus. However, there were no changes in the intensity of RNA foci or CUG_exp_ transcript levels, only a variable trend toward a reduction in CUG_exp_ RNA in the MBNL1 cohort. Similar effects on the stability or turnover of CUG_exp_ RNA foci have been observed with MBNL1 binding decoys (31); however, the mechanism for this effect is unclear. While the MBNL isoforms used for overexpression were predominant at the mRNA level in mouse and human heart tissues, the reduction of CUG_exp_ RNA foci might be due to the isoform of MBNL1 protein that is expressed.

Some previous studies reported that overexpression of MBNL proteins can have a deleterious effect in WT mice (31, 39, 40). A recent study generated mice with heart-specific overexpression of MBNL1 induced at 8 to 10 weeks of age or with constitutive heart-specific expression (22). The results indicated that the timing of MBNL overexpression determines whether there are toxic effects (22). The transgenic mice with early expression displayed both systolic and diastolic dysfunction and concentric hypertrophy and had fewer cardiomyocytes compared with their control littermates. However, MBNL1 overexpression at 8–10 weeks of age, after cardiomyocytes have exited the cell cycle, did not have detrimental effects (22). In our study, we overexpressed MBNL1 and/or MBNL2 proteins starting at 8–9 weeks of age for up to 5 months and observed no heart function or morphology defects in any of the cohorts. Transcriptomics analysis of atrial and ventricular RNA revealed differential expression and alternative splicing changes in several genes involved in the regulation of cardiac action potential and calcium handling, as well as in genes related to cardiomyopathy and arrhythmia. Importantly, most of these genes were significantly, yet partially, rescued with MBNL overexpression. These data identified candidates for future work to study the unknown molecular mechanisms underlying the conduction deficits and functional and structural changes in DM1 hearts. While the focus has been on misregulated alternative splicing, DM1 cardiac features could result from combinations of misregulated alternative splicing and gene expression. For example, the *Scn5a* gene undergoes a splicing switch to more inclusion of the fetal exon 6A, as well as a reduction in total mRNA expression levels in both DM1 and CUG960 +dox heart tissue (27, 41) (Figure 5, E and F, Figure 6E, and Supplemental Figure 9, E and F). Studies from our laboratory and others found that forced expression of the fetal isoform in adult mouse hearts led to cardiac conduction defects and arrhythmias consistent with a role in DM1 cardiac pathology (42–44). However, we recently demonstrated that forced expression of the adult isoform does not rescue cardiac defects in CUG960 +dox mice (41). The results indicate a role for decreased Scn5a expression in DM1 cardiac pathogenesis, given that SCN5A loss of function in humans causes a variety of cardiac defects and that *Scn5a^+/–^* mice show heart conduction defects (45–48).

In conclusion, we demonstrated that heart-specific overexpression of MBNL1 and/or MBNL2 proteins in CUG960 +dox mice substantially rescued multiple physiological, cellular, and molecular aspects of cardiac phenotypes caused by CUG_exp_ RNA expression. Importantly, we found that exogenous overexpression of MBNL1 and MBNL2 that was at least 2-fold above endogenous levels only resulted in approximately 50% rescue of the molecular and physiological effects of CUG_exp_ RNA. Our findings suggest that additional factors participate in cardiac pathogenesis induced by CUG_exp_ RNA. Last, this study recapitulates the near-complete reversal of the cardiac phenotypes in the DM1 cardiac mouse model after shutdown of CUG_exp_ expression, which emphasizes the importance of CUG_exp_ RNA as a therapeutic target for DM1. A study of reversibility following long-term CUG_exp_ RNA expression in the heart will be of particular relevance to the human disease.

## Methods

### Sex as a biological variable.

Our study examined both male and female animals, and similar findings are reported for both sexes.

### Mouse husbandry and transgenic mice.

All mice were housed, maintained, and kept on a 12-hour light/12-hour dark cycle in the animal facilities at Baylor College of Medicine.

TREDT960I/TREDT960I; MHC-rtTA (CUG960) mice were previously established (27). TREDT960I mice (stock no. 032050) were obtained from The Jackson Laboratory. MHC-rtTA–transgenic mice [FVB/N-Tg(Myh6-rtTA)8585Jam/Mmmh; RRID: MMRRC_010478-MU] were originally obtained from Mutant Mouse Resource and Research Centers (MMRRC) (28). In short, TREDT960I/TREDT960I; MHC-rtTA/MHC-rtTA mice were mated with TREDT960I/TREDT960I mice to obtain F1 progeny of experimental CUG960 mice. The study consisted of both male and female animals. Mice were provided dox-containing chow (2 g dox/kg chow, Bioserv) beginning at P1 through nursing dams. Genomic DNA was isolated from tail clips using DirectPCR lysis reagent (Viagen Biotech) and evaluated by PCR using transgene-specific primers for genotypes obtained from MilliporeSigma (Supplemental Table 3). The expected band size for the TREDT960I allele was 331 bp, and the expected band size for the MHC-rtTA allele was 265 bp.

### Cloning of MBNL1, MBNL2, and control constructs.

pAAV-TNNT2-FLAG-MBNL1-TNNT2-MYC-MBNL2, pAAV-TNNT2-3XFLAG-MBNL1-WPRE-SV40pA, pAAV-TNNT2-3XMYC-MBNL2-WPRE-SV40pA, pAAV-TNNT2-mCherry-WPRE-SV40pA, and pAAV-TNNT2-tdTomato-WPRE-SV40pA were generated by commercial DNA synthesis and conventional cloning. Plasmids and complete plasmid sequences are available upon request. All final AAV constructs underwent inverted terminal repeat (ITR) screening using plasmid mapping (restriction enzyme XmaI, SnaBI, and PvuII) and sequencing to confirm that the ITRs were still intact.

### RT-qPCR and RNA splicing.

Total RNA was isolated from atrial and LV tissues from 21-week-old mice using the RNeasy Fibrous Tissue Mini Kit (QIAGEN) according to the manufacturer’s instructions. Homogenization was carried out using a Bullet Blender 2.4 Tissue Homogenizer (Next Advance) with 0.1 g of 0.5 mm diameter Zirconium Oxide Beads (Next Advance). Random-primed cDNA was prepared from 1 μg total RNA using the High-Capacity cDNA Reverse Transcription Kit (Applied Biosystems, 4368813).

RT-qPCR was conducted using PowerUp SYBR-Green PCR Master Mix (Applied Biosystems) and the CFX Connect Real-time system (Bio-Rad). All samples were analyzed in triplicate, and expression levels of *DMPK*, *Scn5A*, *Ryr2*, *Asph*, *Kcna4*, *Cdnf*, *Ckmt2*, *Gja5*, and *Hcn4* were normalized to those of *Rpl4*. Relative expression levels were determined by the 2^–ΔΔCt^ method. Primers for *DMPK*, *Scn5A*, *Ryr2*, *Asph*, *Kcna4*, *Cdnf*, *Ckmt2*, *Gja5*, *Hcn4*, and *Rpl4* were obtained from MilliporeSigma (Supplemental Table 4).

For analysis of alternative splicing events, primers annealing to flanking constitutive exons were designed and obtained from MilliporeSigma (Supplemental Table 5). RT-PCR was performed on cDNA using AmfiSure PCR Master Mix (GenDepot). PCR products were run on a 5% polyacrylamide gel (0.1 M Tris, 0.1 M boric acid, 2 mM EDTA, 5% acrylamide/bis, 19:1 [Bio-Rad], 0.1% ammonium persulfate [APS], 0.18% tetramethylethylenediamine [TEMED] [Bio-Rad]) in TBE running buffer (0.1 M Tris, 0.1 M boric acid, 2 mM EDTA)). The gels were then incubated in 0.4 μg/mL ethidium bromide in TBE for 10 minutes and imaged using Kodak Gel Logic 2200 and Carestram software. Percent spliced in (PSI) values were calculated using densitometry using the following equation: PSI = 100 × [inclusion band/(inclusion band + skipping band)].

### Protein extraction and Western blotting of mouse tissues.

Tissue protein extracts were prepared from atrial and LV heart tissues from 21-week-old mouse by homogenization in 1× RIPA lysis buffer (Cell Signaling Technology, 9806) containing 1× Halt protease and phosphatase inhibitor cocktail (Thermo Fisher Scientific, 78440). The tissues were mixed with RIPA buffer and 0.10 g of 1.0 mm diameter Zirconium Oxide Beads (Next Advance) and blended with the Bullet Blender 2.4 Tissue Homogenizer (Next Advance) followed by sonication. Cellular debris was cleared by centrifugation (15,000 rpm for 15 minutes at 4°C). Protein concentrations of the supernatant were measured using the Pierce BCA Protein Assay Kit (Thermo Fisher Scientific). Samples were diluted and prepared in 6× Laemmli SDS sample buffer (Alfa Aesar, J61337). The samples were then boiled for 5 minutes and 30 μg protein lysates was run on 4%–20% Tris-glycine SDS-PAGE gels (Bio-Rad, 5678095) in Tris/Glycine/SDS Running Buffer (Bio-Rad). The proteins were subsequently transferred onto Trans-Blot Turbo Nitrocellulose Membranes (Bio-Rad) using the Trans-Blot Turbo system (Bio-Rad) at 2.5 A, 25 V for 7 minutes in Trans-Blot Turbo Transfer Buffer (Bio-Rad). Total protein was visualized by Ponceau S staining, and the membranes were washed with PBS (10 mM Na_2_HPO4, 2.7 mM KCl, 137 mM NaCl, 1.76 mM KH_2_PO4, pH 7.4) with 0.01% Tween-20 (MilliporeSigma) (PBST) for 5 minutes. After blocking for 40 minutes at room temperature with 5% Blotto Non Fat Dry-Milk (Chem Cruz) in PBST, THE membranes were probed with anti-MBNL1 (in-house antibody made by Bethyl Laboratories, 1:4,000 dilution), anti-MBNL2 (Santa Cruz Biotechnology, sc-136167, 1:1,000 dilution), anti-FLAG (MilliporeSigma, F1804, 1:4,000 dilution), anti-Myc (MilliporeSigma, 05-724, 1:1,000 dilution), anti-RFP (Proteintech, 6G6, 1:2,000 dilution), or anti-vinculin (MilliporeSigma, V9131, 1:5,000 dilution) primary antibodies in PBST overnight at 4°C. The membranes were washed 3 times in PBST and incubated for 1 hour at room temperature in HRP-conjugated goat anti-rabbit or goat anti-mouse antibodies (Jackson ImmunoResearch, 1:10,000 dilution) in 1% nonfat milk in PBST. After washing 3 times in PBST, the blots were incubated in SuperSignal West Femto Maximum Sensitivity Substrate (Thermo Fisher Scientific, PI34096) for 3 minutes and then imaged on a ChemiDoc XRS+ Imaging system (Bio-Rad).

### Echo.

To assess in vivo cardiac function and morphology, Echo was performed using a Vevo F2 ultrasound machine equipped with a 40 MHz transducer-UHF57x (FujiFilm Visualsonics). Mice were anesthetized with 2% isoflurane, and the chest hair was removed 2 days before conducting Echo. For imaging, mice were subjected to 2% isoflurane and 1.5% oxygen while being placed on the heated Rodent Surgical Monitor+ platform (Indus Instruments), and their body temperature was maintained between 36.5°C and 37.5°C. M-mode images were acquired in the short-axis position at the level of the papillary muscles for each animal. Three M-mode tracings were analyzed per animal using the Visualsonics VevoLab analysis package, and values were averaged.

### ECG.

Data on ECGs were obtained using a multichannel amplifier followed by conversion to digital signals for analysis. Surface ECG recordings were measured from 3 leads using a Rodent Surgical Monitor+ platform (Indus Instruments), and data were acquired using PowerLab software (AD Instruments). Mice were anesthetized using 2% isoflurane and 1.5% oxygen in an induction chamber and were kept under the same condition for data collection with the Rodent Surgical Monitor+ platform (Indus Instruments). Body temperature was monitored using a rectal probe and LabChart software (AD Instruments) and maintained between 36.5°C and 37.5°C. Data were collected for 1.5–2 minutes per mouse and analyzed using LabChart software (AD Instruments). The QTc interval was calculated using Bazett’s formula (49).

### FISH-IF.

Hearts were placed in 20% sucrose (MilliporeSigma, 84097) in 1× nuclease-free PBS (Thermo Fisher Scientific, AM9625) until sinking and were then frozen in OCT medium (Sakura Finetek, 4583) chilled on dry ice. Combined FISH-IF was performed on 7 μm frozen cardiac sections, which were fixed with 4% paraformaldehyde (Alfa Aesar, 43368) in 1× PBS (GenDEPOT, P2101-050, pH 7.4) for 30 minutes at room temperature and washed 5 times with 1× PBS for 2 minutes each, followed by permeabilization in 2% prechilled acetone (Fisher Chemical, A949-1) in 1× PBS for 10 minutes. Sections were incubated with 30% formamide (MilliporeSigma, S4117) in 2× saline sodium citrate (Invitrogen, Thermo Fisher Scientific, AM2616) (SSC) for 30 minutes, hybridized with Cy5-labeled (CAG)5 locked nucleic acid probes (QIAGEN) overnight at 56°C in hybridization buffer (30% formamide, 2XSSC, 0.02% BSA, 66 μg/mL yeast tRNA (Invitrogen, Thermo Fisher Scientific, AM7119), and 2 mM vanadyl ribonucleoside complex (MilliporeSigma, R3380-5ML) and washed for 30 minutes with 30% formamide in 2× SSC at 56°C. Sections were washed in 1× SSC for 30 minutes at room temperature followed by overnight incubation at 4°C in mouse monoclonal anti-MBNL1 (in-house antibody made by Bethyl Laboratories, 1:50 dilution), anti-MBNL2 (3B4, Santa Cruz Biotechnology, sc-136167, 1:50 dilution), anti-FLAG (M2, MilliporeSigma, F1804, 1:50 dilution), or anti–Myc Tag (clone 4A6, Alexa Fluor 555 conjugate, MilliporeSigma, 16-225, 1:500 dilution) antibodies in blocking buffer including 0.1% Triton-X (MilliporeSigma) and 5% BSA (Thermo Fisher Scientific, Fisher Bioreagents, BP1600-100) in 1× PBS. Sections were washed 3 times in 1× PBS for 5 minutes each washing, incubated in Alexa Fluor 488–labeled goat anti-mouse secondary antibody (Invitrogen, Thermo Fisher Scientific, 1:1,000), and washed 3 times with 1× PBS for 5 minutes each washing. The sections were incubated with DAPI (0.5 μg/mL) for 10 minutes and washed with 1× PBS 3 times for 5 minutes each washing. The sections were incubated with 0.1% Sudan black B (MilliporeSigma, 199664-25G) in 70% ethanol for 7 minutes at room temperature, washed 3 times with 1× PBS for 5 minutes each washing, and mounted in Prolong Glass Antifade Mountant (Thermo Fisher Scientific, P36984) with Precision Cover Glasses (1.5 H Thickness, Thorlabs, CG15KH).

Samples were imaged on a Zeiss LSM880 with Airyscan FAST Confocal Microscope with a 63×/1.4 PlanApo oil objective. Four fields of view per section were acquired using laser lines at 405 nm, 488 nm, and 633 nm. Images were processed using Fiji software with CUG960 +dox with the AAV9-tdTomato cohort as a negative control for FLAG and MYC signals, as well as CUG960 –dox as a negative control for foci signals. Using a custom CellProfiler pipeline, the nuclei were segmented, and foci were subsequently identified within the nuclear mask and filtered by 10 minimum and 200 maximum pixel units using positive (CUG960 +dox with AAV-tdTomato cohort) and negative (CUG960 –dox cohort) controls for reference. Mean foci counts per nucleus were determined for each individual nuclear mask and averaged across all fields of view. Foci intensity was determined using pixel intensity data measured by the Zeiss LSM880 with the Airyscan FAST Confocal Microscope, analyzed using the CellProfiler pipeline described above to quantify the mean intensity of each individual nuclear foci, and subsequently averaged across all fields of view.

### RNA-Seq analysis.

RNA was isolated from the atria and left ventricles using the RNeasy Fibrous Tissue Mini Kit (QIAGEN). Members of the Genomic and RNA Profiling Core (GARP) at Baylor College of Medicine conducted sample quality checks using the NanoDrop spectrophotometer (Thermo Fisher Scientific) and the Agilent Bioanalyzer 2100 (Agilent Technologies). Samples that met the following criteria were used for RNA-Seq: RNA integrity number (RIN) of 8.0 or higher, A260 nm/A280 nm of 1.9 or higher, A260 nm/A230 nm of 1 or higher, and r28S/16S of 1.5 or higher. The Illumina TruSeq Stranded mRNA library preparation protocol was then used to generate cDNA libraries starting with 250 ng total RNA. The resulting libraries were quantitated using the NanoDrop spectrophotometer, and fragment size was assessed with the Agilent Bioanalyzer. The pooled libraries were loaded onto a NovaSeq 6000 S4 flow cell and sequenced to a depth of approximately 100 million read pairs/sample. A paired-end, 150-cycle run was used to sequence the flowcell on a NovaSeq 6000 sequencing system.

The quality of raw fastq files was first assessed using FastQC, version 0.11.9 (http://www.bioinformatics.babraham.ac.uk/projects/fastqc/). Raw reads were then mapped with STAR, version 2.7.10b (50), using the mm10 genome and the GENCODE M25 gene annotation in GFF3 format (51). Raw read counts were processed and normalized to transcripts per million (TPM) using the RSEM algorithm, version 1.3.1 (52) DGE was called with an adjusted *P* value of less than 0.05 and an absolute fold change of 1.5 or more using DESeq2, version1.42.0 (53). For alternative splicing (AS) analysis, rMATS , version 4.1.2 (54), was applied to identify and quantify 5 different splicing types: CE, alternative 5′ splice site (AS 5′ SS), alternative 3′ splice site (AS 3′ SS), mutually exclusive exons (MXEs), and retained intron (RI) events, based on the bam files generated from the STAR alignment. Splicing events with reads of 20 or higher, an FDR of 0.05 or less, and a ΔPSI of 0.15 or more between 2 conditions were considered significant AS events. RNA-Seq data for DGE and AS can be found in the Supplemental Data Files 1–4.

### Signature scores and gene ontology for DGE and AS.

To measure the signature scores using AS events, significant splicing events were identified using the following criteria: FDR of 0.05 or less and an absolute ΔPSI of 0.15 or greater. The significant splicing events in the CUG960 –dox vs. CUG960 +dox group were selected as the AS signatures and included in the score calculation. For each AS signature, the score weights were assigned on the basis of their PSI direction change. Events with a positive PSI change (IncLevelDifference >0 in the CUG960 –dox vs. CUG960 +dox group) received a weight of +1, while events with a negative PSI change (IncLevelDifference <0 in the CUG960 –dox vs. CUG960 +dox group) received a weight of –1. Next, the Virtual Inference of Protein activity by Enriched Regulon (VIPER) analysis (version 1.36.0) algorithm integrated these weighted events and used a rank-based method to compute an integrated score.

Similar to signature scores for AS, to measure the signature scores for DGEs, we used the VIPER R package (version 1.36.0) with the log_2_-transformed transcripts per million (TPM) gene expression matrix as input. The regulons used in the VIPER analysis were the DGEs with an adjusted *P* value of less than 0.05 and an absolute fold change of 1.5 or greater.

ShinyGO V0.80 (55) was used for gene ontology analysis. Enriched categories with a –log_10_ (FDR) ≥1.5 (enrichment FDR ≤ 0.032) were considered significant.

### Statistics.

All quantitative experiments had at least 3 independent biological replicates. Results are presented as the mean ± SEM. Statistical analyses for data sets were carried out using GraphPad Prism, version 9.0 (GraphPad Software), and the methods used are specified in the figure legends. Statistical tests included 1-way ANOVA followed by Tukey’s test for multiple comparisons and 2-way ANOVA followed by Tukey’s or Dunnett’s test for multiple comparisons. A P value of less than 0.05 was considered statistically significant.

### Study approval.

All mouse experiments were carried out in accordance with the *Guide for the Care and Use of Laboratory Animals* (National Academies Press, 2011) and approved by the IACUC of Baylor College of Medicine.

### Data availability.

Data are available in the main text or the supplemental materials. Values for all data points in the graphs are reported in the Supporting Data Values file. The mouse RNA-Seq data have been deposited in the NCBI’s Gene Expression Omnibus (GEO) database (GEO GSE283998).

## Author contributions

RCH and TAC conceived the study, designed experiments, and analyzed and interpreted data. RCH performed the assays and wrote the manuscript. RCH and TAC edited the manuscript. YZ and ZX performed data analysis for RNA-Seq. LN, SJJ, AEH, and WRL helped with the experimental design and data analysis. All authors reviewed the manuscript and provided input.

## Supplementary Material

Supplemental data

Supplemental data set 1

Supplemental data set 2

Supplemental data set 3

Supplemental data set 4

Unedited blot and gel images

Supporting data values

## Version 1 02/11/2025

In-Press Preview

## Version 2 04/01/2025

Electronic publication
