# Supplementary material for: MBNL overexpression rescues cardiac phenotypes in a myotonic dystrophy type 1 heart mouse model
Source: J Clin Invest. 2025 Feb 11;135(7):e186416. doi: 10.1172/JCI186416 (PMC11957708; doi:10.1172/JCI186416)
Supplement: Supplemental data [file jci-135-186416-s091.pdf]

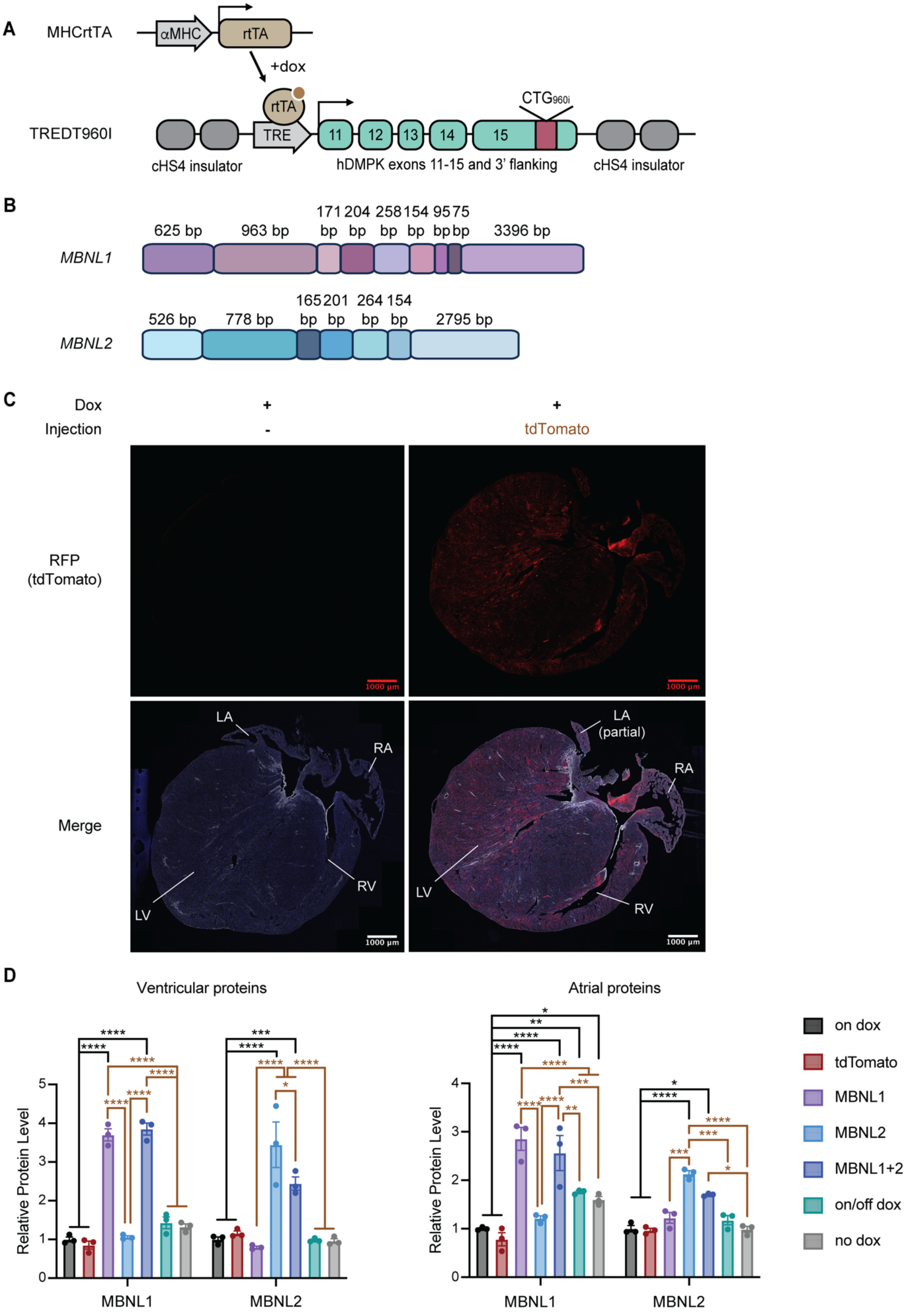
A
MHCrtTA
αMHC
rtTA
+dox
TREDT960I
rtTA
TRE
11
12
13
14
15
CTG960i
cHS4 insulator
hDMPK exons 11-15 and 3' flanking
cHS4 insulator
B
MBNL1
625 bp
963 bp
171 bp
204 bp
258 bp
154 bp
95 bp
75 bp
3396 bp
MBNL2
526 bp
778 bp
165 bp
201 bp
264 bp
154 bp
2795 bp
C
Dox
Injection
tdTomato
RFP (tdTomato)
Merge
LA
RA
RV
LV
LA (partial)
1000 μm
D
Ventricular proteins
Atrial proteins
Relative Protein Level
MBNL1
MBNL2
on dox
tdTomato
MBNL1
MBNL2
MBNL1+2
on/off dox
no dox

Supplemental Figure 1. Exogenous cardiac MBNL1 and MBNL2 isoforms successfully overexpressed in the heart of the CUG960 DM1 heart bitransgenic mouse model. (**A**) Schematic of the TREDT960I/MHCrtTA (CUG960) heart mouse model. TRE: tetracycline response element; rtTA: reverse tetracycline transactivator. (**B**) Diagram of the predominant cardiac MBNL1 and MBNL2 isoforms, shown as mRNAs, used for overexpression in this study. bp: base pair (**C**) Immunofluorescence detection for tdTomato in whole heart of CUG960 +dox control mice and AAV9-tdTomato treated mice using anti-red fluorescent protein (RFP) antibody to check for the distribution of AAV9 virus. Blue signal represents DAPI and grey signal represents wheat germ agglutinin (WGA). Data shown here are tile a scan image; scale bar is 1000 μm. LV: left ventricle; RV: right ventricle; LA: left atrium; RA: right atrium. (**D**) Quantification data for MBNL1 and MBNL2 protein levels from western blots in Figure 1C normalized to Vinculin. n=3 animals per cohort. Data represent the means ± SEM and were analyzed using 2-way ANOVA followed by Dunnett's multiple comparisons test. * $p<0.05$, ** $p<0.01$, *** $p<0.001$, **** $p<0.0001$. Black lines represent the significant differences of corresponding groups compared to +dox or tdTomato controls. Brown lines represent the significant differences between corresponding groups and +/-dox and -dox controls.

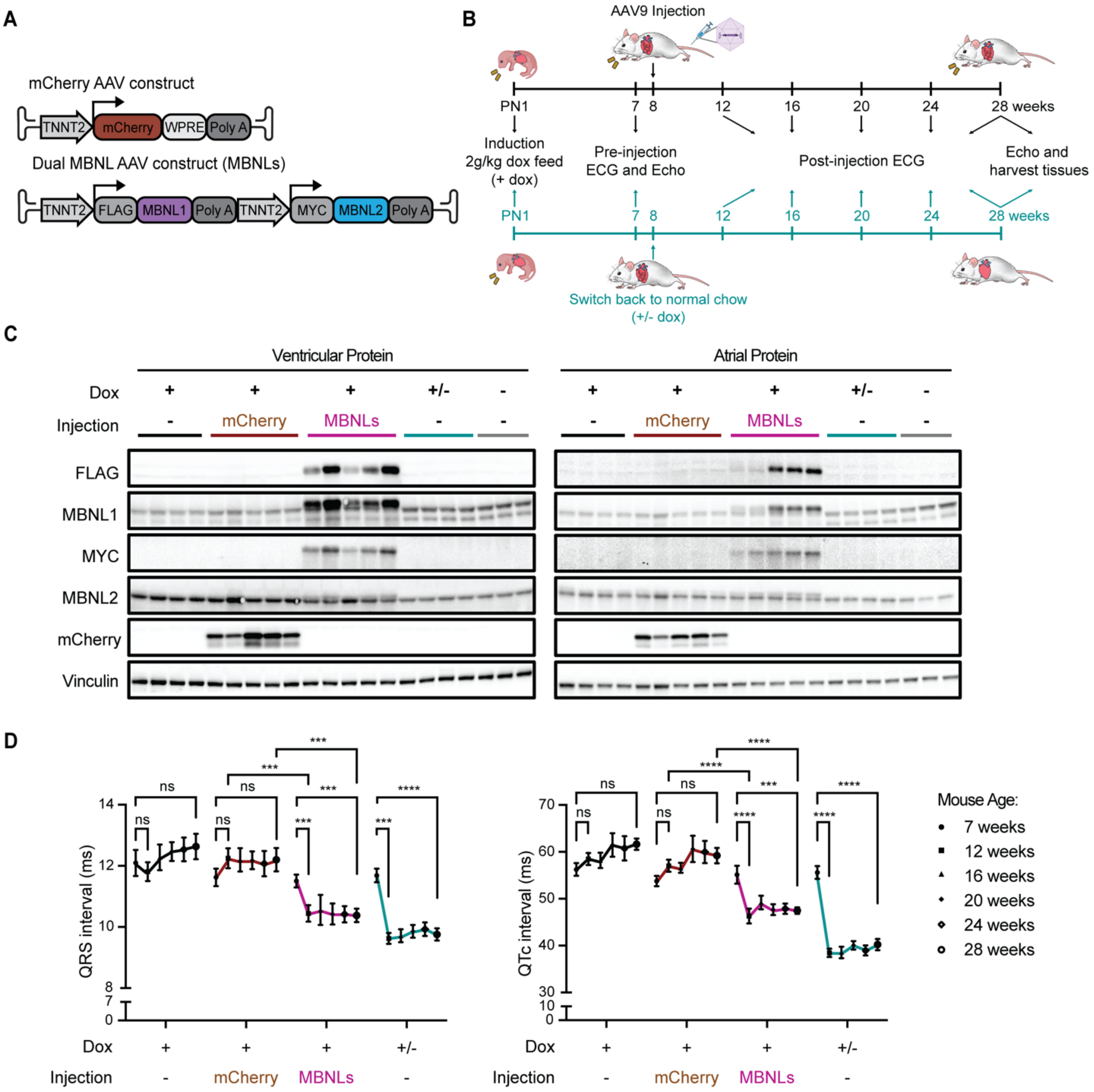

Supplemental Figure 2. Rescue of conduction defects by MBNL overexpression remained consistent for 5 months after injection. (**A**) Diagram of the pAAV vectors used to express FLAG-MBNL1-MYC-MBNL2 (pAAV-MBNL1&2) and mCherry protein (pAAV-mCherry) as a control. (**B**) Diagram of the experimental plan including timepoints and assays. (**C**) Cardiac ventricular and atrial protein expression were evaluated by western blotting analysis. (**D**) QRS (left) and QTc (right) intervals determined by surface ECG recordings in anesthetized CUG960 mice in response to dox induction with different treatments. *n*=9 per cohort. Data represent the mean ± SEM and were analyzed using Mixed-effects analysis followed by Tukey's multiple comparisons test. * $p<0.05$, *** $p<0.001$, **** $p<0.0001$, ns: not significant.

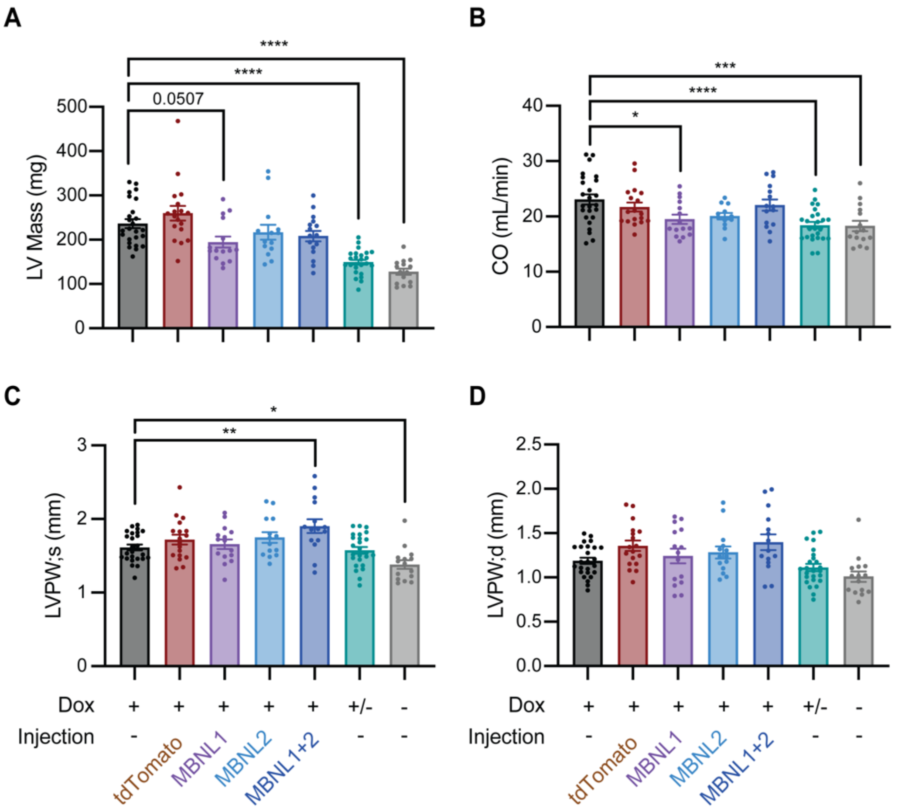

Supplemental Figure 3. M-mode echocardiography was performed on animals from all cohorts at 21-weeks of age to evaluate (**A**) LV mass, (**B**) cardiac output (CO), (**C** and **D**) left ventricular posterior wall (LVPW) thickness. d: end of diastole. s: end of systole. $n \geq 13$ per cohort. Data represent the mean ± SEM and were analyzed using ordinary one-way ANOVA. * $p<0.05$, ** $p<0.01$, *** $p<0.001$, **** $p<0.0001$.

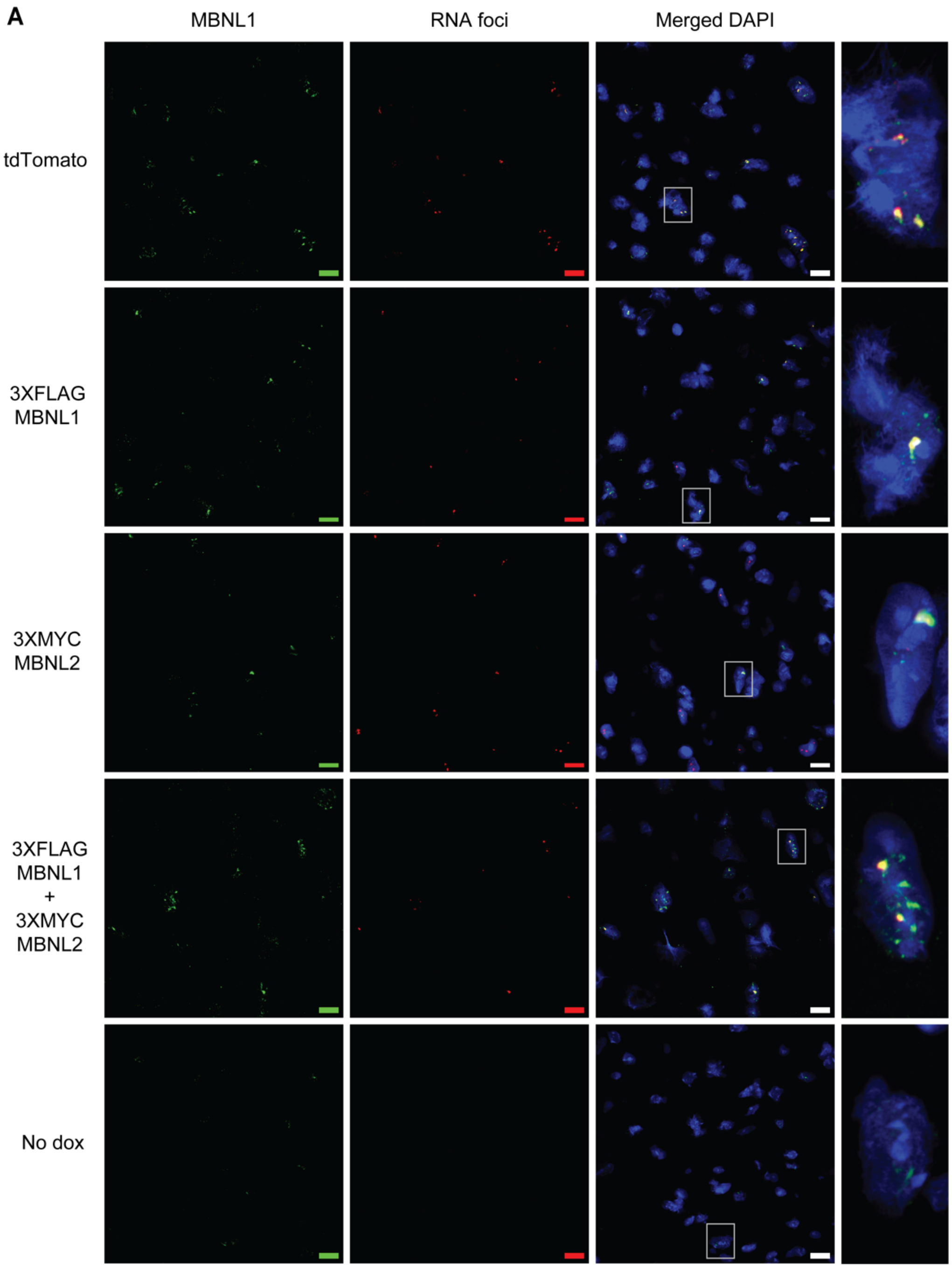
A
MBNL1
RNA foci
Merged DAPI
tdTomato
3XFLAG
MBNL1
3XMYC
MBNL2
3XFLAG
MBNL1
+
3XMYC
MBNL2
No dox

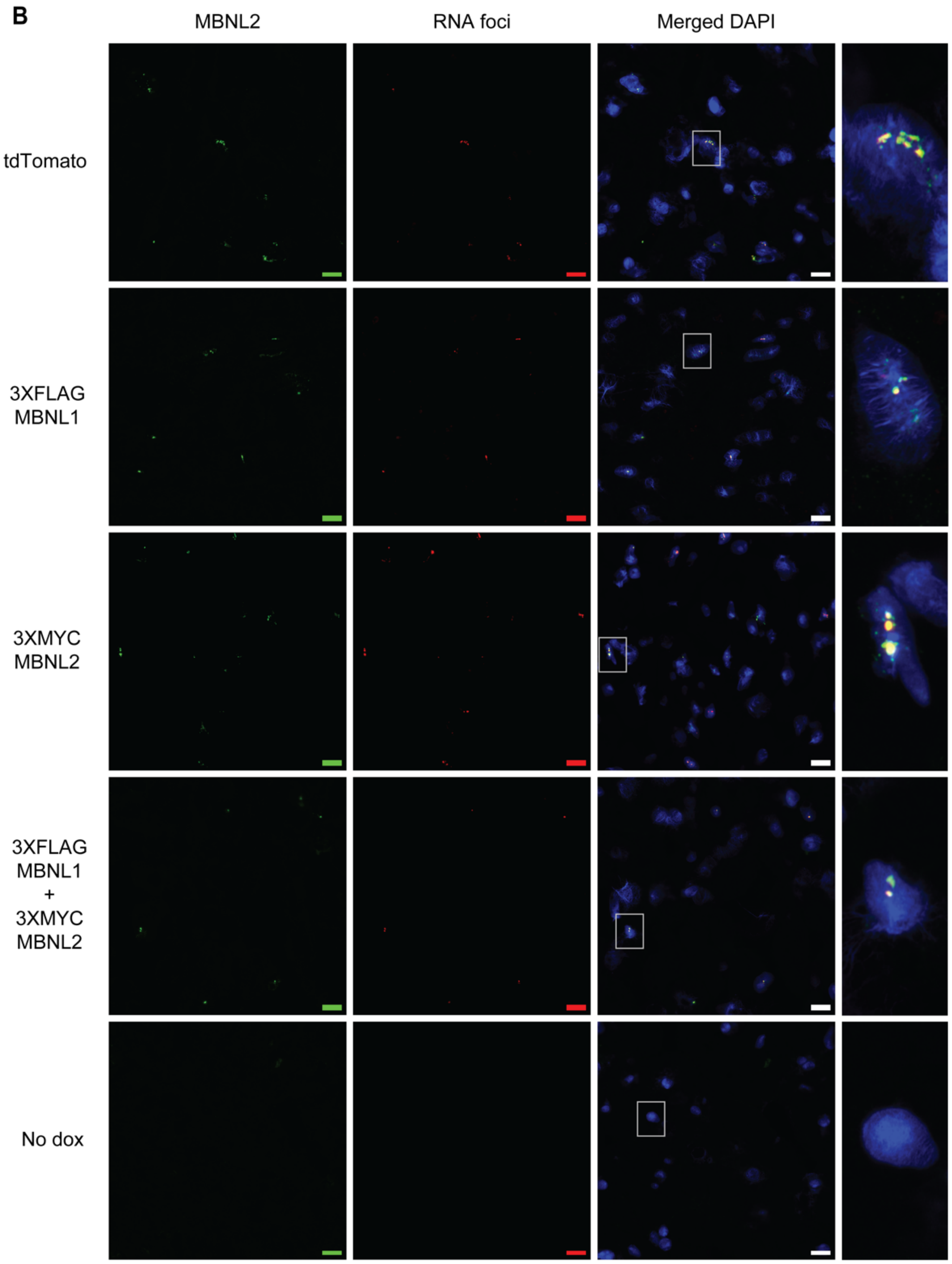

Supplementary Figure 4. CUG960 +dox mice with MBNL overexpression showed MBNL1 and MBNL2 colocalized with the foci. RNA FISH with a $(CAG)_5$ locked nucleic acid probe targeting CUG_{exp} RNA combined with immunofluorescence for (**A**) MBNL1 and (**B**) MBNL2 in the ventricles of CUG960 +dox mice. The experiments were conducted on three animals for each group and the representative images are shown. Scale bar: 10 microns.

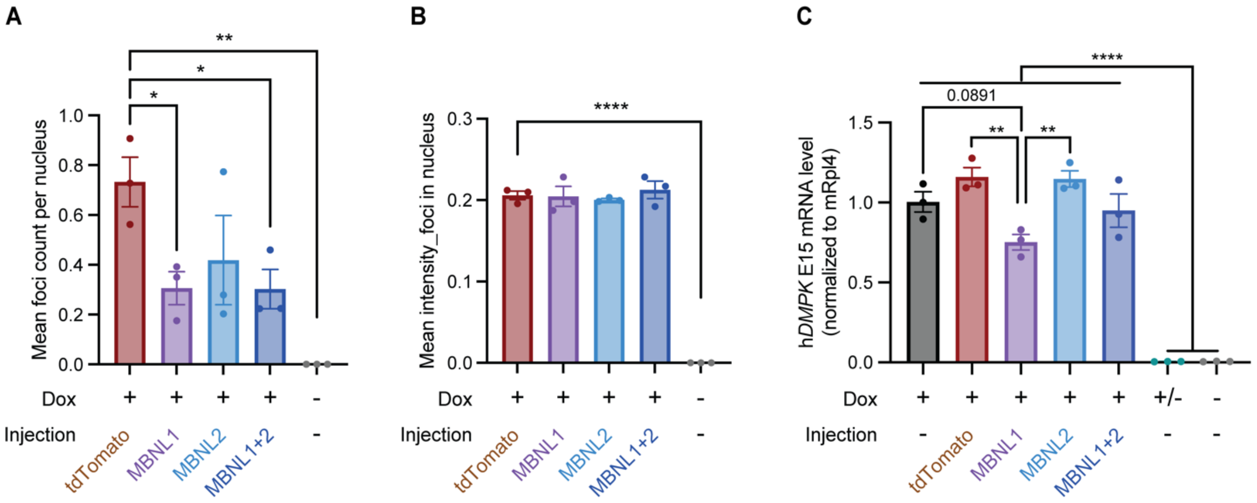

Supplementary Figure 5. Overexpression of MBNL decreased the number of foci but not the expression of CUG_{exp} RNA repeats in ventricles. (**A**) Total number of nuclear foci divided by the total number of nuclei measured in 3 ventricular samples from each cohort. Four fields of view were analyzed per animal and 3 ventricular samples from 3 different animals per group were used. (**B**) Quantification of foci intensity for foci in nucleus from each cohort. (**C**) RT-qPCR analysis of transgene mRNA expression in ventricle of CUG960 mice in response to different conditions. m*Rpl4* was used as an internal control for normalization. *n*=3 animals per cohort. Data represent the mean ± SEM and were analyzed using ordinary one-way ANOVA followed by Dunnett's multiple comparisons test. * $p<0.05$, ** $p<0.01$, **** $p<0.0001$.

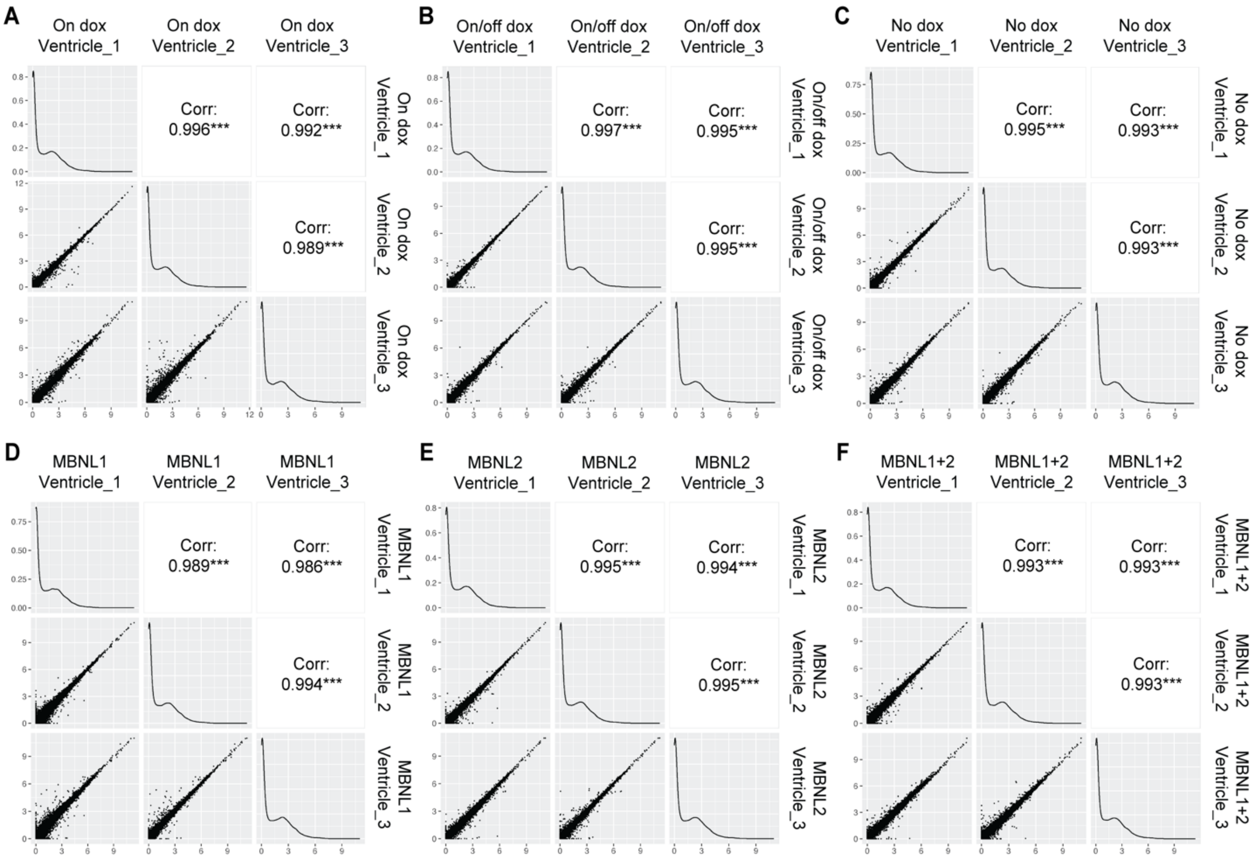

Supplemental Figure 6. Reproducibility between biological replicates in ventricular gene expression. Biological replicate RNA-seq data sets for ventricles analyzed for reproducibility in gene expression. The X and Y-axis represent the normalized transcript per million (TPM) values and each dot means a gene.

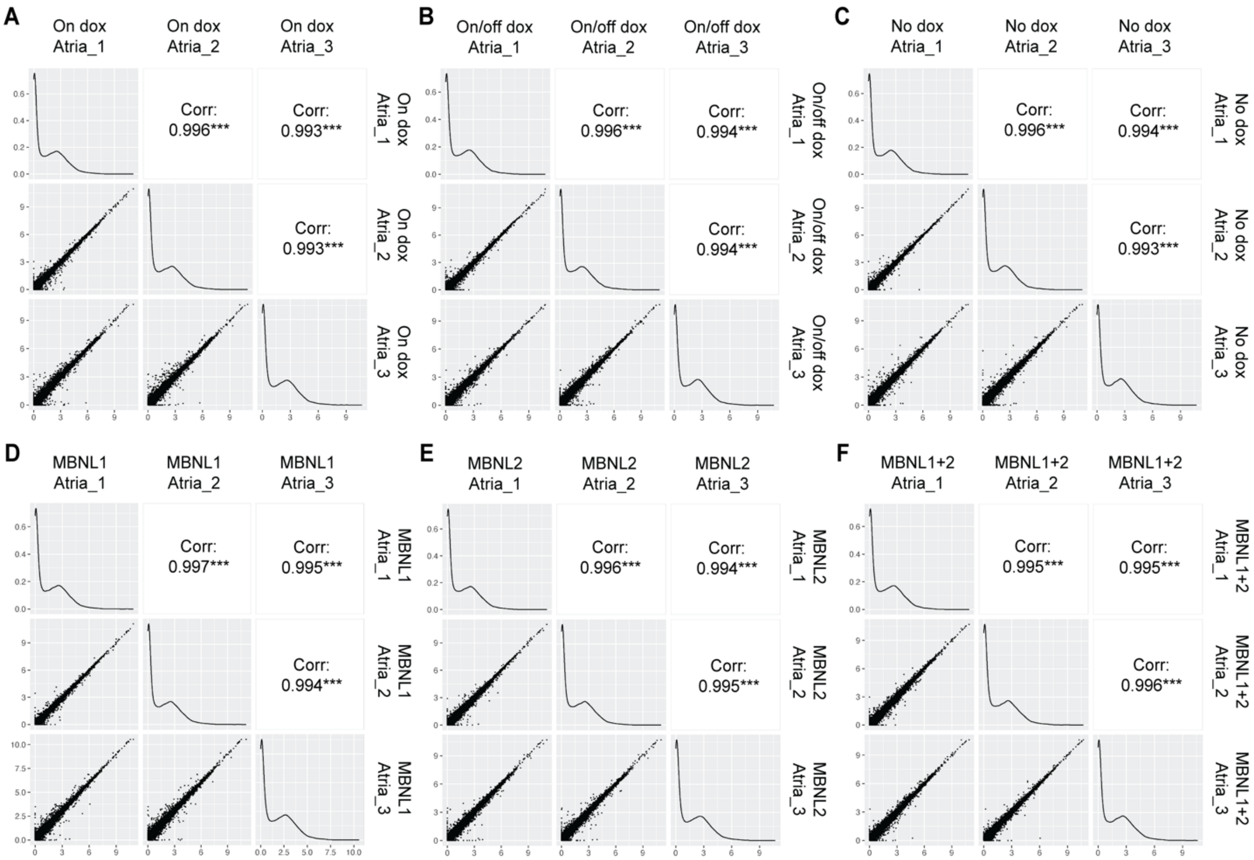

Supplemental Figure 7. Reproducibility between biological replicates in atrial gene expression. Biological replicate RNA-seq data sets for atria analyzed for reproducibility in gene expression. The X and Y-axis represent the normalized transcript per million (TPM) values and each dot means a gene.

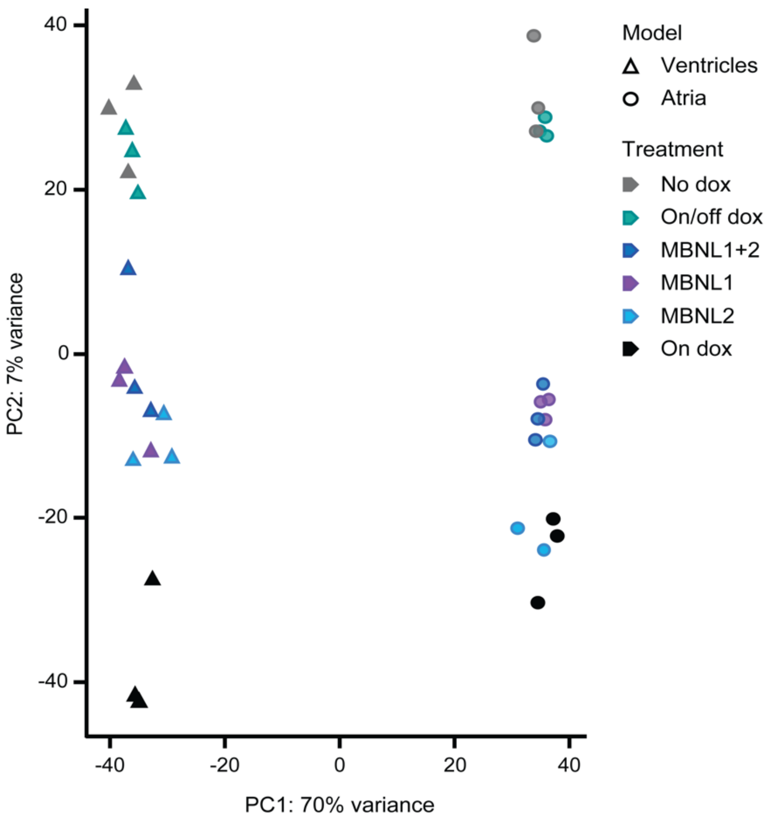

Supplementary Figure 8. PCA showed strong differences between ventricular and atrial samples, as well as separation of different cohorts.

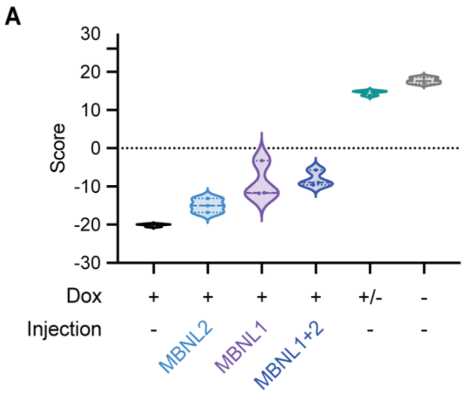

B

Treatments compared to +dox group	Alternative splicing (AS)				
	A3SS	A5SS	MXE	IR	CE
AAV9-MBNL1	9	9	41	0	185
AAV9-MBNL2	7	4	18	1	95
AAV9-MBNL1+2	12	8	47	2	234
on/off dox	16	16	122	2	538
no dox	20	19	112	2	554

C

Comparisons	Total CE	Overlapping CE	Percentage
+/- dox vs +dox	538	538	100%
MBNL1+2 vs +dox	234	171	31.8%
MBNL1 vs +dox	185	132	24.5%
MBNL2 vs +dox	95	56	10.4%

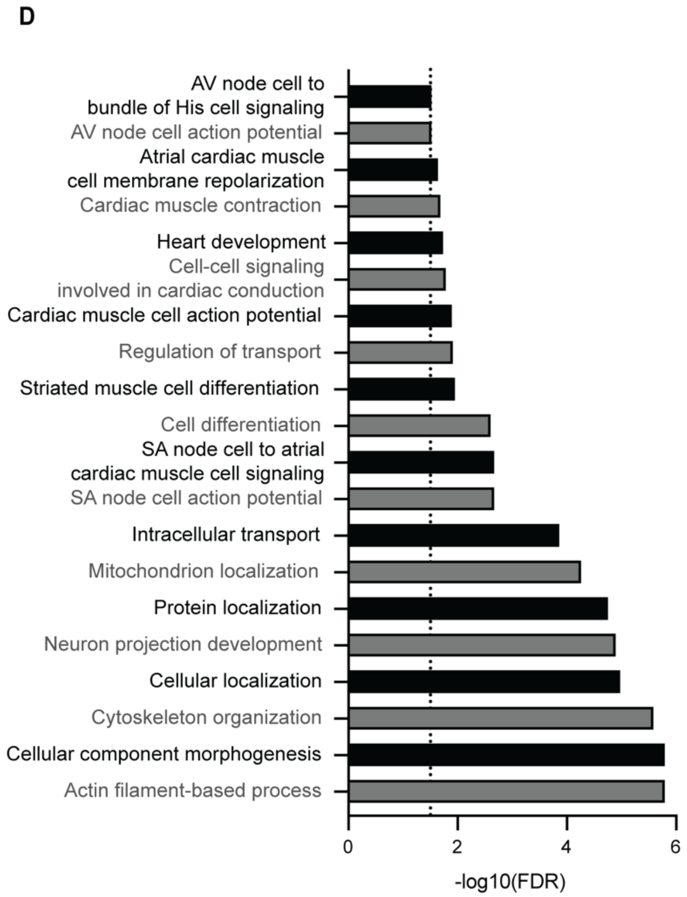

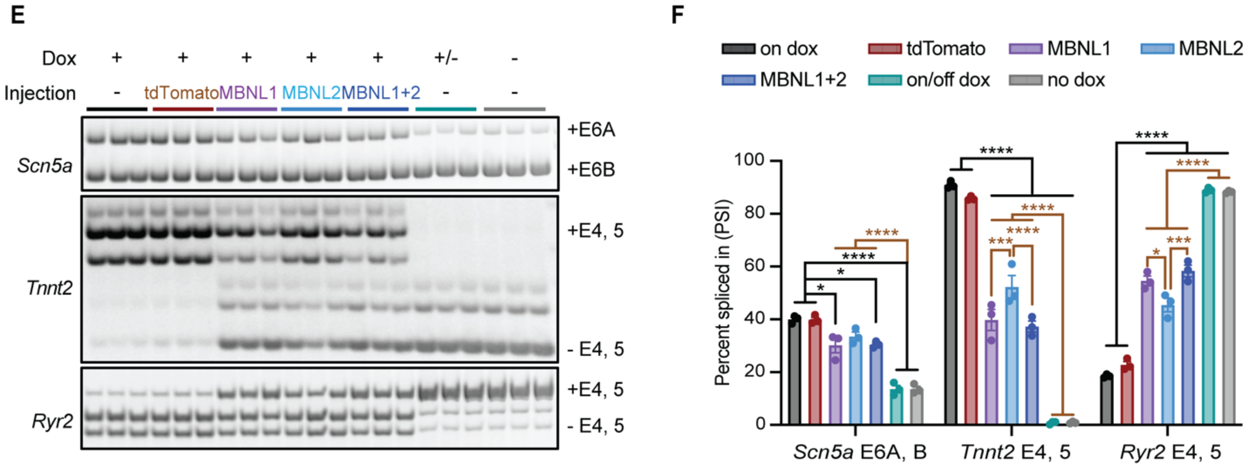

Supplemental Figure 9. MBNL overexpression in the heart leads to rescues of disrupted transcriptomic-wide splicing events related to cardiac morphology and function in atria of CUG960 +dox mice. (**A**) Alternative splicing signature score of atrial samples. The central line represents the median value, while the upper and lower dashed lines indicate the first and third quartiles. (**B**) Numbers and types of alternative splicing events observed in atria. A3SS/A5SS: alternative 3'/5' splice site; MXE: mutually exclusive exons; IR: intron retention; and CE: cassette exons. (**C**) Percentage of overlapping cassette exon splicing events in each MBNL overexpression group using the comparison between on/off dox and on dox as reference. (**D**) Genes showing

differential alternative splicing changes in comparisons of CUG960 -dox to +dox mice, as well as in comparisons of all MBNL cohorts to CUG960 +dox mice were evaluated for enrichment of gene ontology functional terms using ShinyGO platform. Dash line: -log10(false discovery rate) = 1.5 (**E**) Representative RT-PCRs showing alternative splicing changes for candidate genes, *Scn5a, Tnnt2, and Ryr2*, in ventricles of CUG960 +dox mice with different treatments. (**F**) Quantification of percent spliced in (PSI) for each splicing events. $n = 3$ animals per cohort. Data represent the mean ± SEM and were analyzed using ordinary two-way ANOVA followed by Tukey's multiple comparisons test. * $p<0.05$, ** $p<0.01$, *** $p<0.001$, **** $p<0.0001$. Black lines represent the significant differences of corresponding groups compared to +dox or tdTomato controls. Brown lines represent the significant differences between corresponding groups and +/-dox and -dox controls. E: exon. Dox: doxycycline. Reg.: regulation.

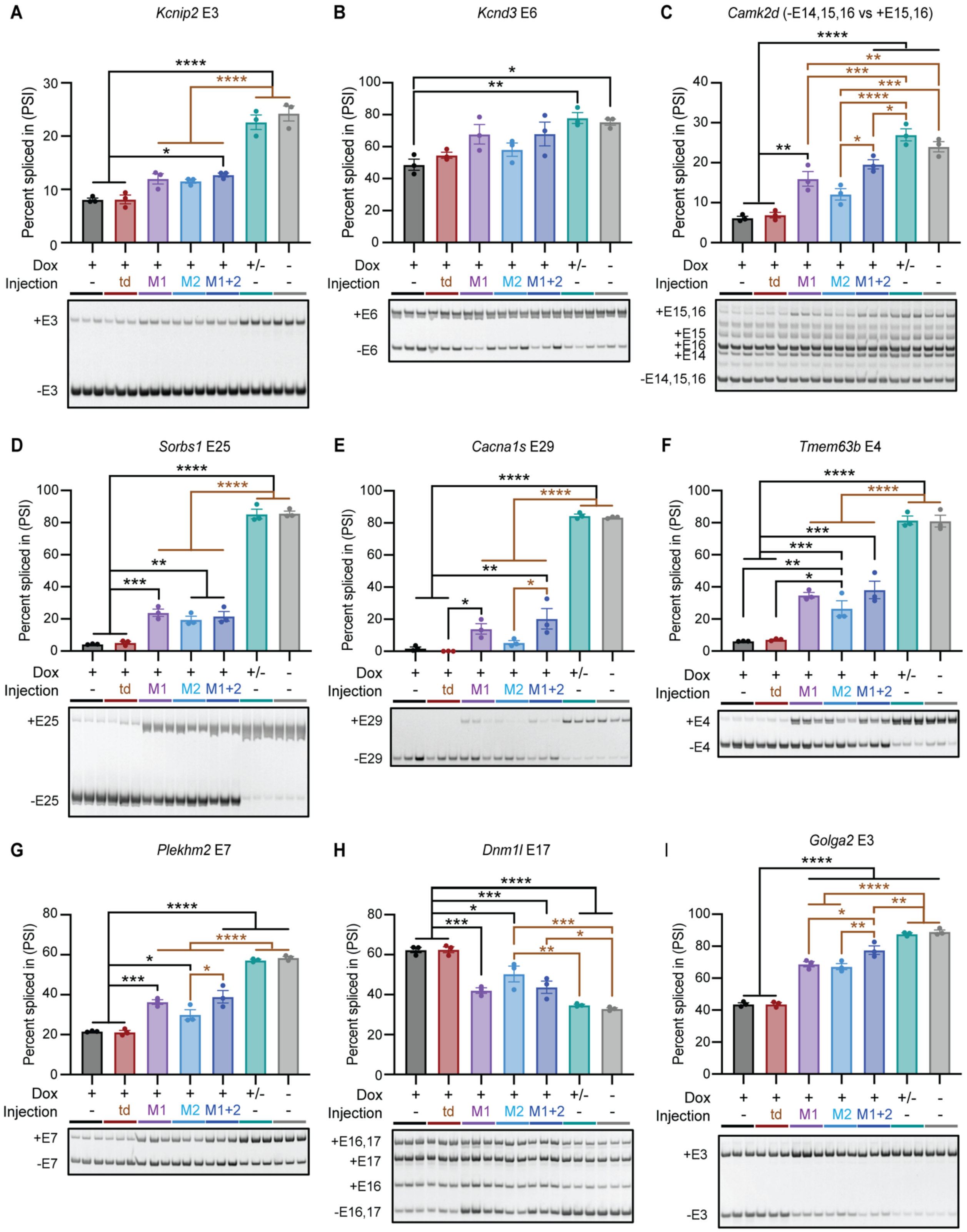

Supplemental Figure 10. Validation of ventricular splicing events. Representative RT-PCRs showing alternative splicing changes and quantification of percent spliced in (PSI) for each

splicing events in ventricles of CUG960 +dox mice with different treatments. *n = 3* animals per cohort. Data represent the mean ± SEM and were analyzed using ordinary one-way ANOVA followed by Tukey's multiple comparisons test. * $p<0.05$, ** $p<0.01$, *** $p<0.001$, **** $p<0.0001$. Black lines represent the significant differences of corresponding groups compared to +dox or tdTomato controls. Brown lines represent the significant differences between corresponding groups and +/-dox and -dox controls. E: exon. Dox: doxycycline. Td: tdTomato. M1: MBNL1. M2: MBNL2. M1+2: MBNL1+MBNL2.

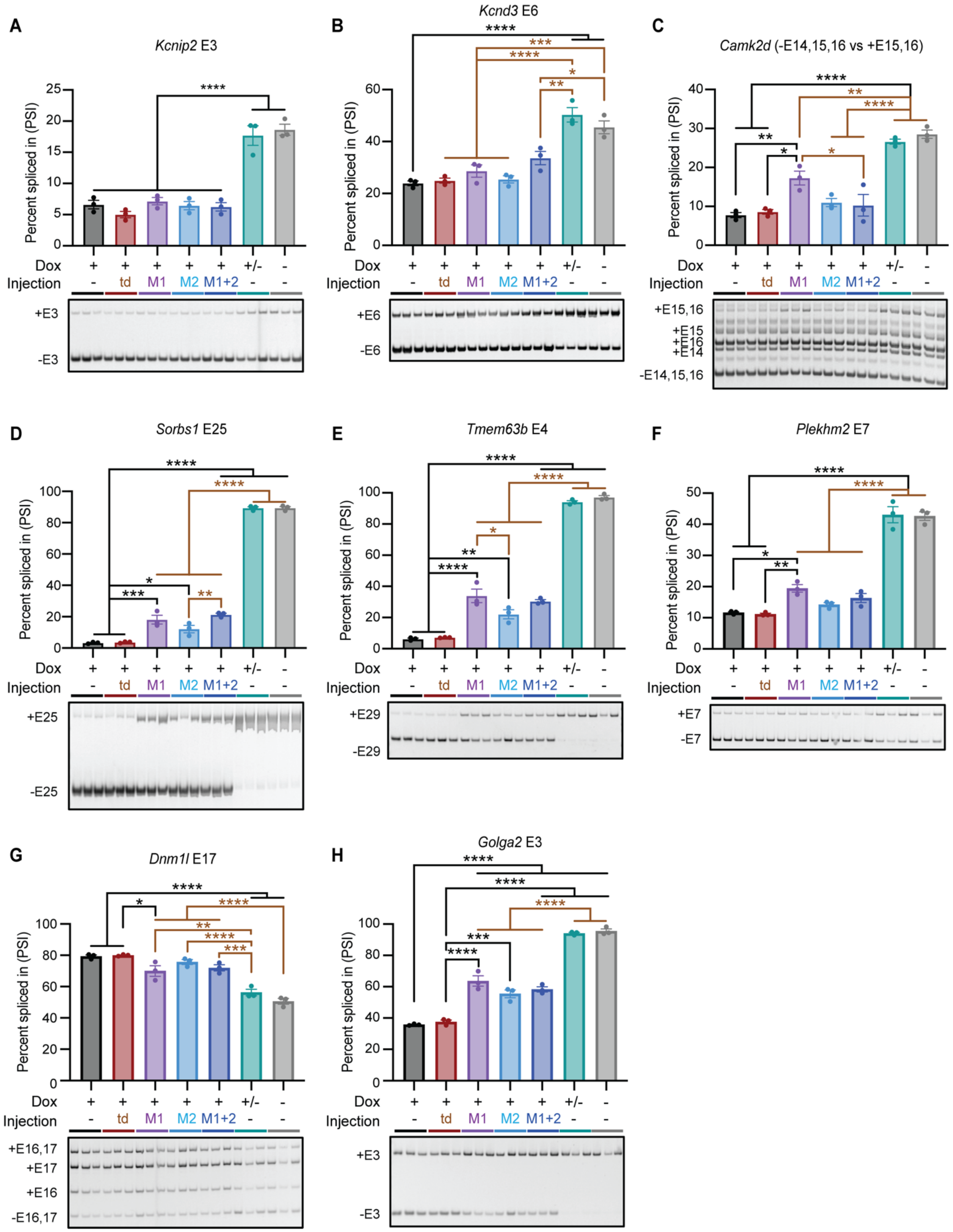

Supplemental Figure 11. Validation of atrial splicing events. Representative RT-PCRs showing alternative splicing changes and quantification of percent spliced in (PSI) for each splicing events

in atria of CUG960 +dox mice with different treatments. $n = 3$ animals per cohort. Data represent the mean ± SEM and were analyzed using ordinary one-way ANOVA followed by Tukey's multiple comparisons test. * $p<0.05$, ** $p<0.01$, *** $p<0.001$, **** $p<0.0001$. Black lines represent the significant differences of corresponding groups compared to +dox or tdTomato controls. Brown lines represent the significant differences between corresponding groups and +/-dox and -dox controls. E: exon. Dox: doxycycline. Td: tdTomato. M1: MBNL1. M2: MBNL2. M1+2: MBNL1+MBNL2.

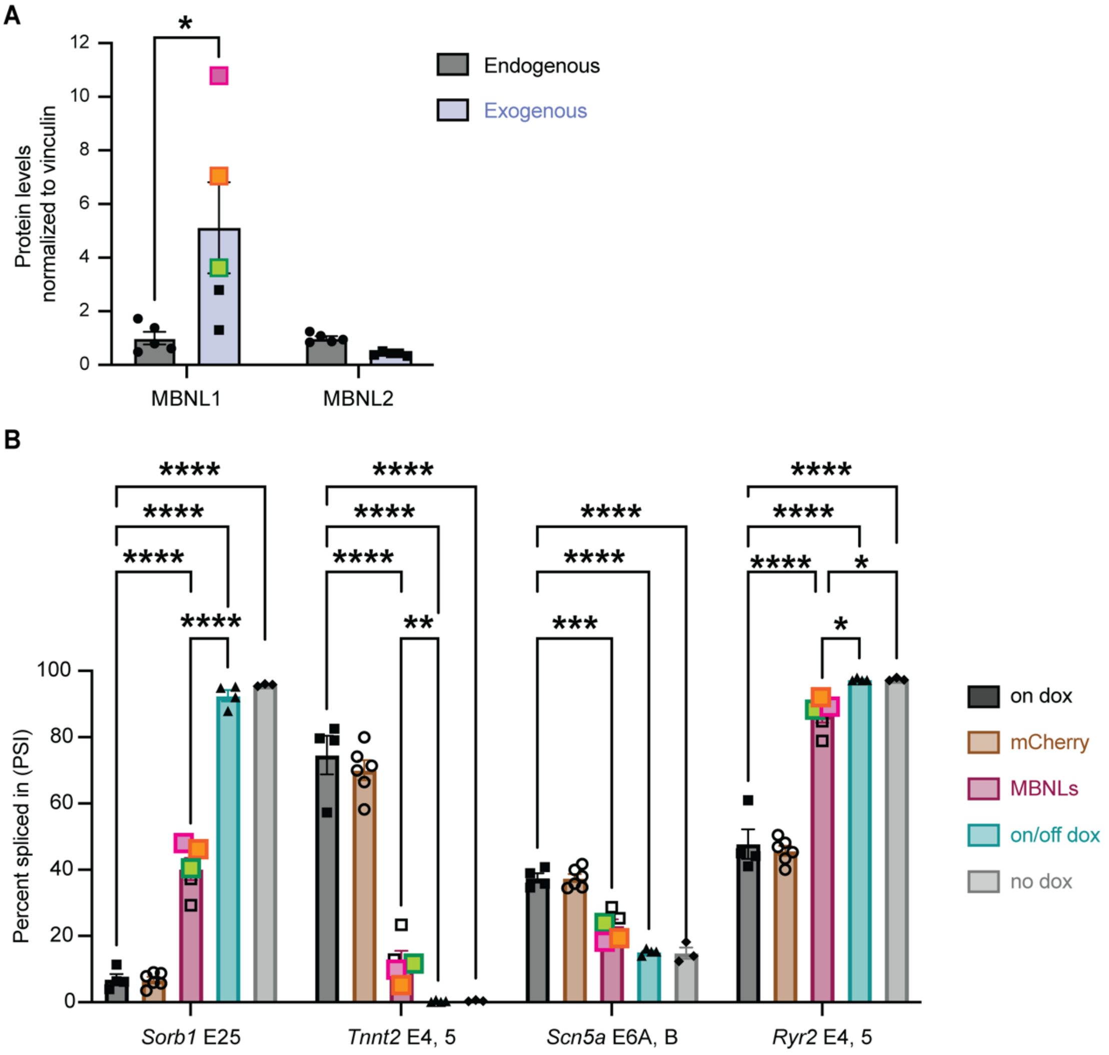

Supplemental Figure 12. Splicing rescue increased only incrementally with increased MBNL1 expression. (**A**) Quantification of MBNL1 and MBNL2 protein levels of AAV-MBNLs cohort from western blots in Supplemental Figure 2C. The protein levels of MBNL1 and MBNL2 were normalized to Vinculin. (**B**) Quantification of percent spliced in (PSI) for each splicing event. $n \geq 3$ animals per cohort. Green=3.6-fold, Orange=7.0-fold, Pink=10.8-fold exogenous MBNL1 expression over endogenous Mbnl1. Data represent the means ± SEM and were analyzed using 2-way ANOVA followed by Tukey's multiple comparisons test. * $p<0.05$, ** $p<0.01$, *** $p<0.001$, **** $p<0.0001$.

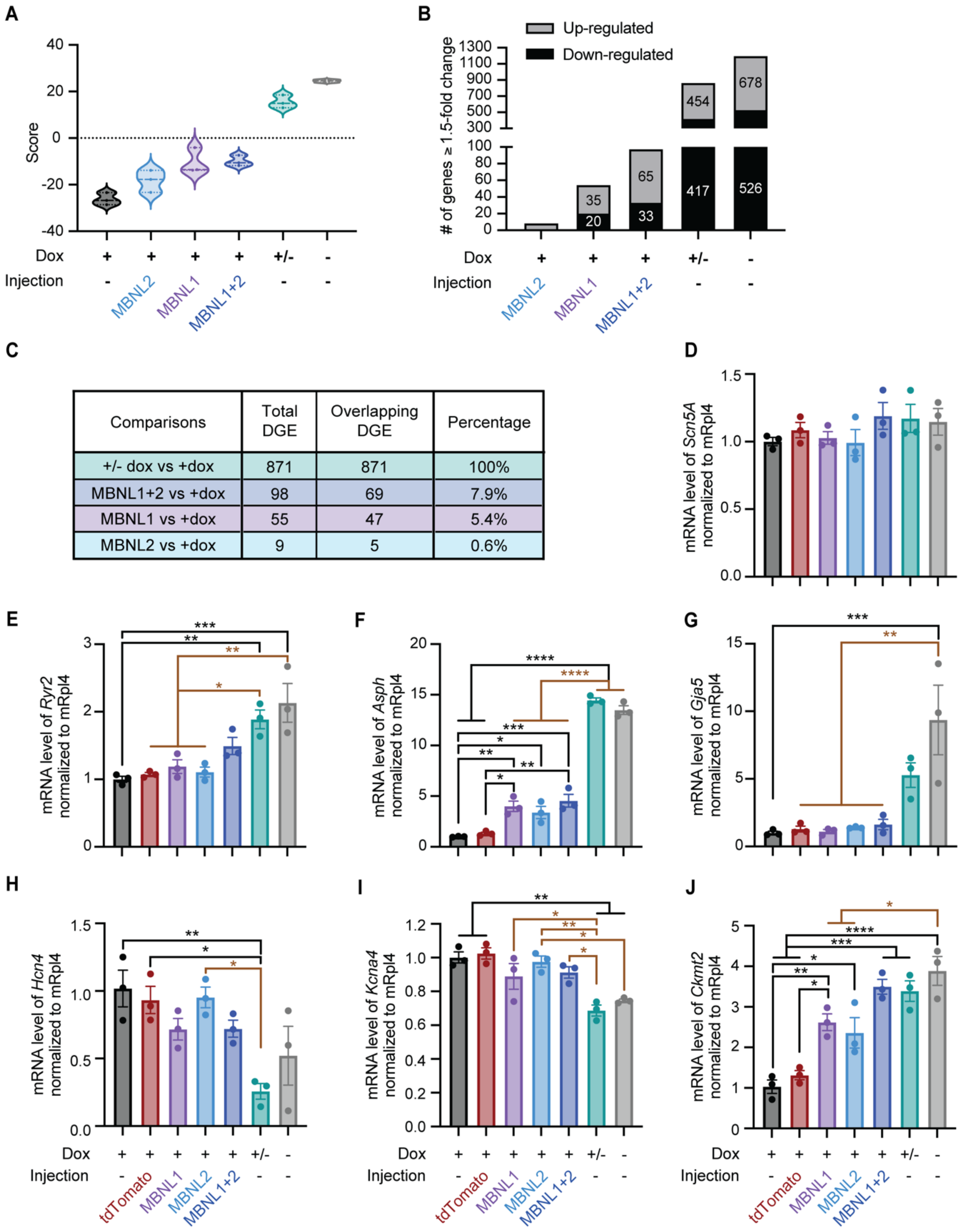

Comparisons	Total DGE	Overlapping DGE	Percentage
+/- dox vs +dox	871	871	100%
MBNL1+2 vs +dox	98	69	7.9%
MBNL1 vs +dox	55	47	5.4%
MBNL2 vs +dox	9	5	0.6%

Supplemental Figure 13. MBNL1 and/or MBNL2 overexpression results in variable differential gene expression (DGE) changes in atria of CUG960 +dox mice. (**A**) Gene signature scores of atrial samples. The central line represents the median value, while the upper and lower dashed lines indicate the first and third quartiles. (**B**) Differentially expressed genes displaying

≥1.5-fold change (*p adj.*≤0.05) as compared to CUG960 +dox controls. For MBNL2 cohort, there are 8 upregulated genes and 1 downregulated gene. (**C**) Percentages of overlapping DGEs in each MBNL overexpression group using the comparison between on/off dox and on dox as reference. (**D-J**) RT-qPCR-based validation of candidate genes showing gene expression changes in atria of each cohort. *Rpl4* mRNA was used as an internal control for normalization. *n*=3 animals per cohort. Data represent the mean ± SEM and were analyzed using ordinary one-way ANOVA followed by Tukey's multiple comparisons test. * $p<0.05$, ** $p<0.01$, *** $p<0.001$, **** $p<0.0001$. Black lines represent the significant differences of corresponding groups compared to +dox or tdTomato controls. Brown lines represent the significant differences between corresponding groups and +/-dox and -dox controls. Dox: doxycycline.

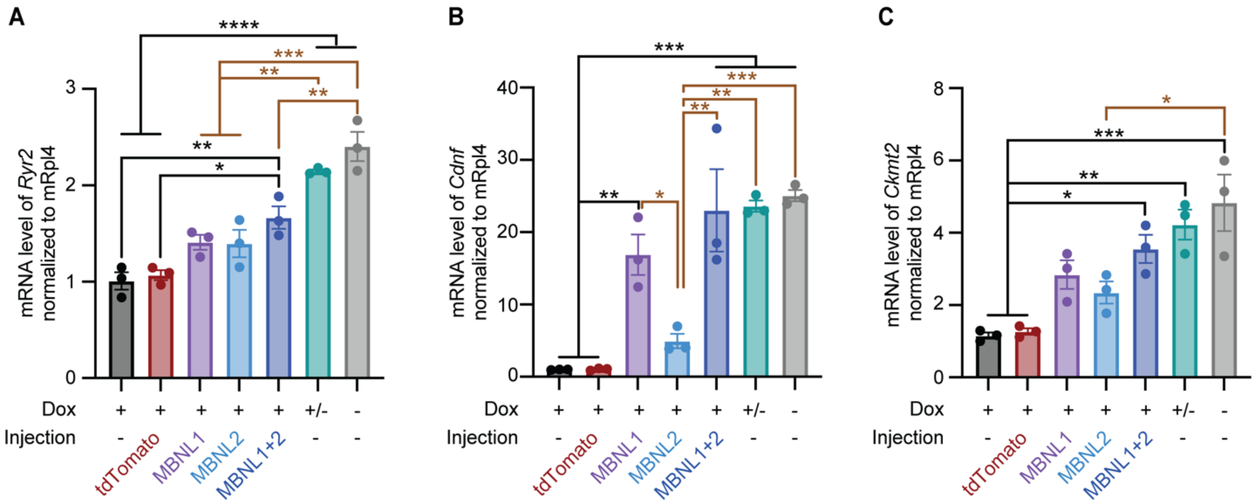

Supplemental Figure 14. RT-qPCR-based validation of candidate genes related to cardiac diseases from ventricle. *Rpl4* mRNA was used as an internal control for normalization. *n*=3 animals per cohort. Data represent the mean ± SEM and were analyzed using ordinary one-way ANOVA followed by Tukey's multiple comparisons test. * $p<0.05$, ** $p<0.01$, *** $p<0.001$, **** $p<0.0001$. Black lines represent the significant differences of corresponding groups compared to +dox or tdTomato controls. Brown lines represent the significant differences between corresponding groups and +/-dox and -dox controls. Dox: doxycycline.

Supplemental Table 1. RNA-sequencing sample information

Sample	Group	Tissue	Sex	Uniquely Mapped Reads	% Reads Uniquely Mapped to Genome
Y620	CUG960 +dox	LV	Male	128375452	66.71%
Y661	CUG960 +dox	LV	Male	127453620	69.86%
Y677	CUG960 +dox	LV	Female	106350478	62.11%
Y628	CUG960 +dox +AAV9-3xflagMBNL1	LV	Male	105188932	63.36%
Y716	CUG960 +dox +AAV9-3xflagMBNL1	LV	Male	102335760	64.46%
Y727	CUG960 +dox +AAV9-3xflagMBNL1	LV	Female	109221514	65.79%
Y610	CUG960 +dox +AAV9-3xmycMBNL2	LV	Male	110352996	63.31%
Y624	CUG960 +dox +AAV9-3xmycMBNL2	LV	Male	107796148	61.92%
Y697	CUG960 +dox +AAV9-3xmycMBNL2	LV	Female	104282427	63.74%
Y858	CUG960 +dox +AAV9-3xflagMBNL1 +AAV9-3xmycMBNL2	LV	Male	98225277	62.60%
Y831	CUG960 +dox +AAV9-3xflagMBNL1 +AAV9-3xmycMBNL2	LV	Male	93139869	59.66%
Y847	CUG960 +dox +AAV9-3xflagMBNL1 +AAV9-3xmycMBNL2	LV	Male	107020400	59.46%
Y607	CUG960 +/-dox	LV	Male	111158075	61.92%
Y608	CUG960 +/-dox	LV	Male	93362626	60.94%
Y725	CUG960 +/-dox	LV	Female	110580008	58.45%
Y762	CUG960	LV	Male	102474518	62.40%
Y764	CUG960	LV	Male	105892978	68.63%
Y752	CUG960	LV	Female	98199813	57.65%
Y620	CUG960 +dox	Atria	Male	119146903	70.00%

Y661	CUG960 +dox	Atria	Male	121460082	68.92%
Y677	CUG960 +dox	Atria	Female	104290909	62.03%
Y628	CUG960 +dox +AAV9-3xflagMBNL1	Atria	Male	112747820	66.44%
Y716	CUG960 +dox +AAV9-3xflagMBNL1	Atria	Male	122189854	68.45%
Y727	CUG960 +dox +AAV9-3xflagMBNL1	Atria	Female	113820876	67.44%
Y610	CUG960 +dox +AAV9-3xmycMBNL2	Atria	Male	115129192	66.35%
Y624	CUG960 +dox +AAV9-3xmycMBNL2	Atria	Male	111244619	65.94%
Y697	CUG960 +dox +AAV9-3xmycMBNL2	Atria	Female	102703000	66.54%
Y858	CUG960 +dox +AAV9-3xflagMBNL1 +AAV9-3xmycMBNL2	Atria	Male	121533954	61.53%
Y831	CUG960 +dox +AAV9-3xflagMBNL1 +AAV9-3xmycMBNL2	Atria	Male	112665766	63.58%
Y847	CUG960 +dox +AAV9-3xflagMBNL1 +AAV9-3xmycMBNL2	Atria	Male	112831031	63.01%
Y607	CUG960 +/-dox	Atria	Male	92174789	58.32%
Y608	CUG960 +/-dox	Atria	Male	106431020	63.75%
Y725	CUG960 +/-dox	Atria	Female	115627232	63.70%
Y762	CUG960	Atria	Male	109753973	65.53%
Y764	CUG960	Atria	Male	106607087	64.20%
Y752	CUG960	Atria	Female	114078725	62.33%

Supplementary Table 2. Gene expressions of different groups compared to CUG960 +dox group from RNA-seq data

Tissue	Gene	Log2FoldChange (compared to CUG960 +dox)				
		No dox	On/off dox	MBNL1	MBNL2	MBNL1+2
Ventricle	*Scn5A*	1.00329467	0.93071661	0.60066553	0.60464806	0.53560179
	Ryr2	0.98166289	1.05298204	0.52699726	0.40108818	0.33759819
	Asph	-0.8273143	-0.8577248	-0.3182677	-0.3314111	-0.3785509
	Kcna4	-2.3238944	-2.3790008	-1.9773778	-1.5326186	-1.0773637
	Ckmt2	1.71456125	1.726045	1.34498804	1.20402091	0.95741393
	Cdnf	3.71095514	3.69325009	3.48374947	3.3540995	1.66681079
Atria	*Ryr2*	0.69755804	0.71764647	NA	NA	NA
	Kcna4	-0.7394501	-0.6110642	-0.5969915	NA	NA
	Ckmt2	1.78867452	1.64349024	1.50629911	1.20004396	1.06935751
	Gja5	2.31732332	1.71754654	NA	NA	NA

Supplementary Table 3. List of primers used for mouse genotyping

Name	Sequence
TREDT960I_F	TGCCGTCGAGTTTACTCCCTATCA
TREDT960I_R	AATTCTCCAGGCGATCTGACGGTT
MHCrtTA_F	CTGGGTTGCGTGTTGGAAGATC
MHCrtTA_R	GTGGGAGATCGAGCAGGCCCTCG

Supplementary Table 4. List of primers used for RT-qPCRs in mouse tissue

Name	Sequence
*hDMPK*_E15_down_F	CCGTGTTCCATCCTCCAC
*hDMPK*_E15_down_R	CCGAGTAAGCAGGCAGAGAT
*mRpl4*_F	CGCTGGTGGTTGAAGATAAGG
*mRpl4*_R	CGGTTTCTCATTTTGCCCTTG
mScn5a_qPCR_F	CTCCAGAGGGTATGAAGAGC
mScn5a_qPCR_R	CCGAAGATGATGAAGACGAC
mRyr2_qPCR_F	ACAGTGGAAAAGTCAGAAGGG
mRyr2_qPCR_R	AGGAATGGCGTAGCAATATGG
*mHcn4*_qPCR_F	CTATGAACACCGCTACCAAGG
*mHcn4*_qPCR_R	CAAAGTTGGGATCTGCGTTG
*mGja5*_qPCR_F	CCTCGGTCTCCTACTCTTGG
*mGja5*_qPCR_R	CATGCGGAAAATGAACAGGAC
*mAsph*_qPCR_F	GGCGATGGAGACTTTGATGT
*mAsph*_qPCR_R	CAGCTCTTCTTTGATGGGTTC
*mCkmt2*_qPCR_F	GAAGGGAGGTGGAGAATGTG
*mCkmt2*_qPCR_R	GGCTTATCAAACAGAAAGTGGTC
*mCdnf*_qPCR_F	GTATCTCGAACCCTGTGCTG
*mCdnf*_qPCR_R	GCTCTTTCTCTATGGTGTCCG
*mKcna4*_qPCR_F	CAGCCAGCTAGAGAGCAAGA
*mKcna4*_qPCR_R	CTGGGAACGGAAGTCAAGGT

Supplementary Table 5. List of primers used for RT-PCRs in mouse tissue

Name	Sequence
*mTnnt2*_E5_F	GTACGAGGAGGAACAGGAAG
*mTnnt2*_E5_R	CCAGCCTCCTCCTCCTCC
*mScn5a*_E6A_F	GTCGGCTCTTCGAACTTTCA
*mScn5a*_E6B_F	CGGGCCCTGAAAACTATATC
*mScn5a*_E7_R	CCAATGAGGGCAAAGACACT
*mRyr2*_E4, 5_F	CGGACCTGTCTATCTGCACCTTTGT
*mRyr2*_E4, 5_R	CATACCACTGTAGGAATGGCGTAGCA
*mSorbs1*_E25_F	CCAGCTGATTACTTGGAGTCCACAGAAG
*mSorbs1*_E25_R	GTTCACCTTCATACCAGTTCTGGTCAATC
*mTmem63b*_E4_F	CTGGCTCTGGACTTCATGTGCTTTC
*mTmem63b*_E4_R	GAGACGGAGGTGAGACGCTCATACC
*mCacna1s*_E29_F	GAGATCCTTGGAATGTGTTTGACTTCCT
*mCacna1s*_E29_R	GGTTCAGCAGCTTGACCAGTCTCAT
*mGolga2*_E3_F	GCAGGAGCAAAGAAGAAGAAAAAGATTAAA
*mGolga2*_E3_R	GGTGCAGGAGCAATATGGTCTGTG
*mPlekhm2*_E7_F	GACAGCCTCTCCCTAAACTCCTTCAAT
*mPlekhm2*_E7_R	GTGGTGTCGGATGATGCAGTCTCT
*mDnm1l*_E16, 17_F	GCCTGTGGGCTAATGAACAATAATATAGA
*mDnm1l*_E16, 17_R	GTTCCTGACCACCGTCTCCAATC
*mKcnip2*_E3_F	CCCTGCCCTCAGTCAGTGAAA
*mKcnip2*_E3_R	TGCGTGTGAACTTGGTTTGTTCC
*mCamk2d*_E14-15-16_F	TGAAGAAACCAGATGGGGTAA
*mCamk2d*_E14-15-16_R	CCTCAAAGTCCCCATTGTTG
*mKcnd3*_E6_F	GGCAAGACCACCTCACTCAT
*mKcnd3*_E6_R	TGGCTGGACAGAGAAGGACT
