# Supplementary material for: MBNL overexpression rescues cardiac phenotypes in a myotonic dystrophy type 1 heart mouse model
Source: J Clin Invest. 2025 Feb 11;135(7):e186416. doi: 10.1172/JCI186416 (PMC11957708; doi:10.1172/JCI186416)

<u>Full unedited blots for Figure 1 (left: with marker; right: image used in figure)</u>

- FLAG (ventricle)

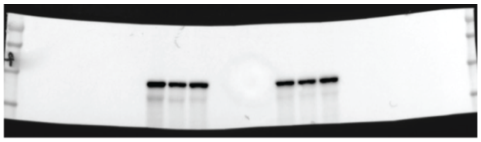

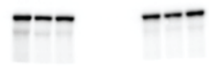

Shown in the figure

- MBNL1 (ventricle)

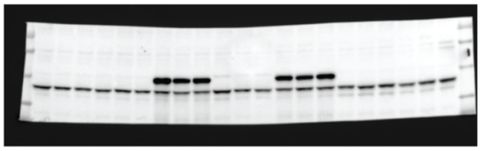

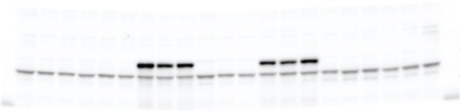

Shown in the figure

- MYC (ventricle)

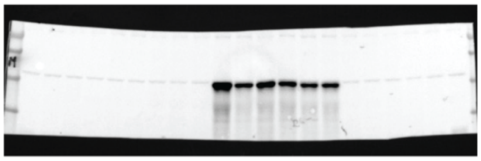

Shown in the figure

- MBNL2 (ventricle)

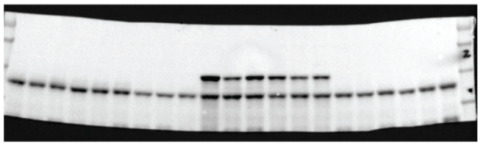

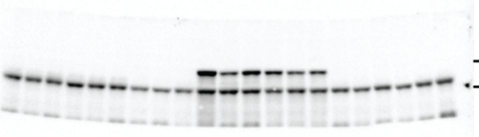

Shown in the figure

- RFP (ventricle; top band was used in the figure)

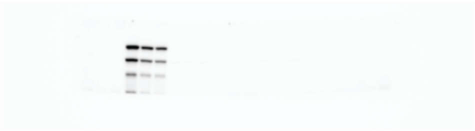

Shown in the figure

- Vinculin (ventricle; the membrane at the bottom was used in the figure)

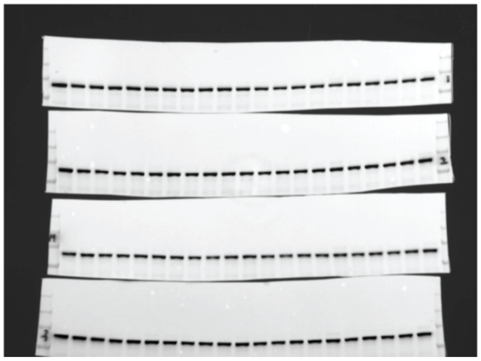

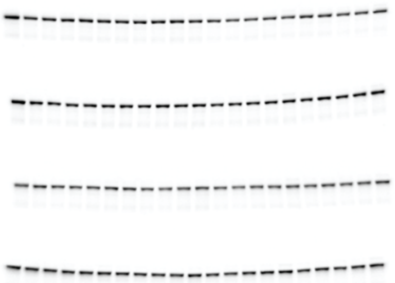

Shown in the figure

- FLAG (atria)

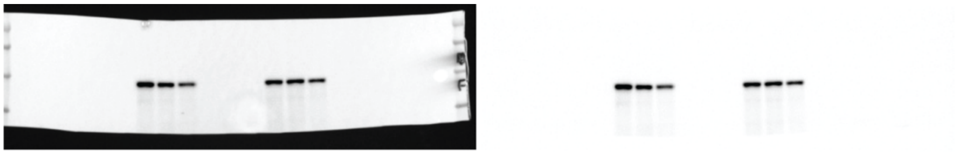

Shown in the figure

- MBNL1 (atria)

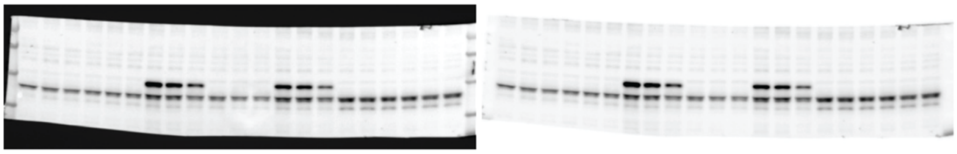

Shown in the figure

- MYC (atria)

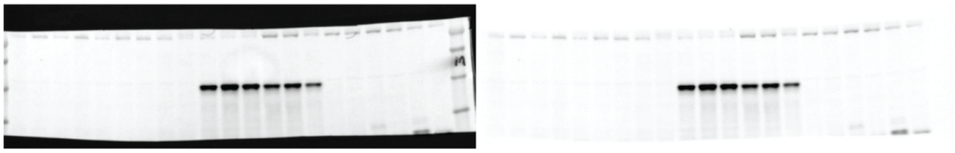

Shown in the figure

- MBNL2 (atria)

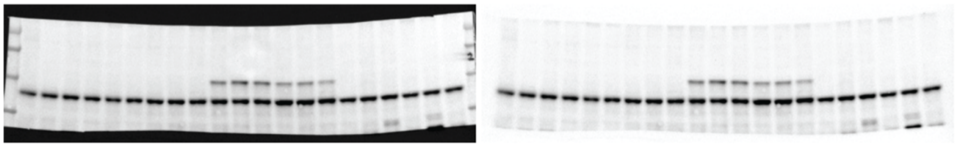

Shown in the figure

- RFP (atria; top band was used in the figure)

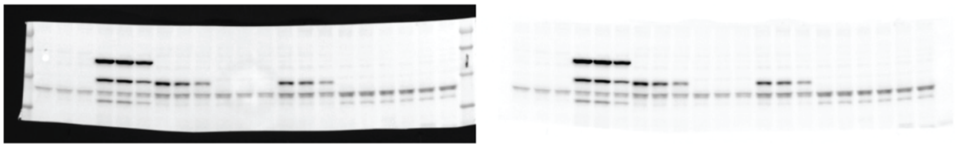

Shown in the figure

- Vinculin (atria; top membrane was used in the figure)

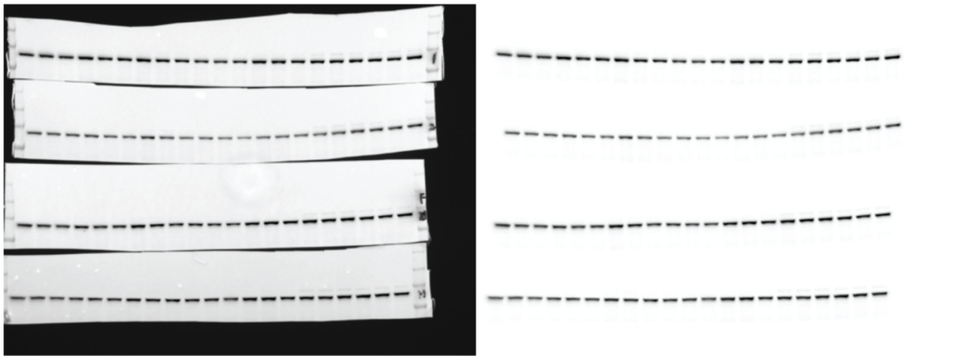

Shown in the figure

<u>**Full unedited gels for Figure 5E and Supplemental Figure 9E**</u>

- Scn5a

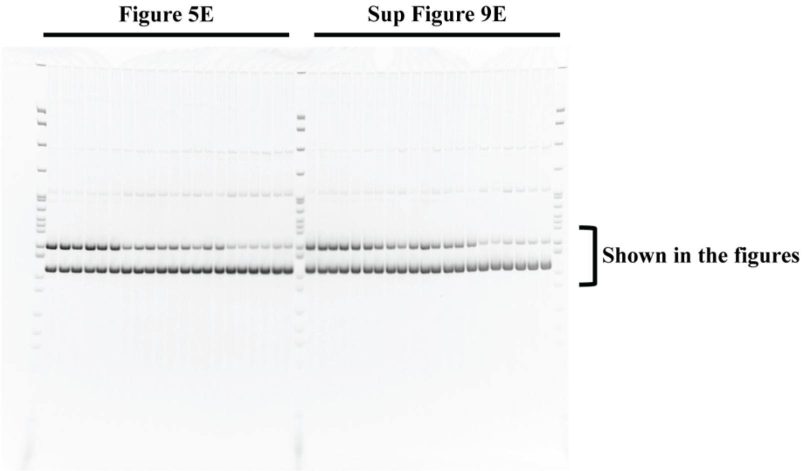

- Tnnt2

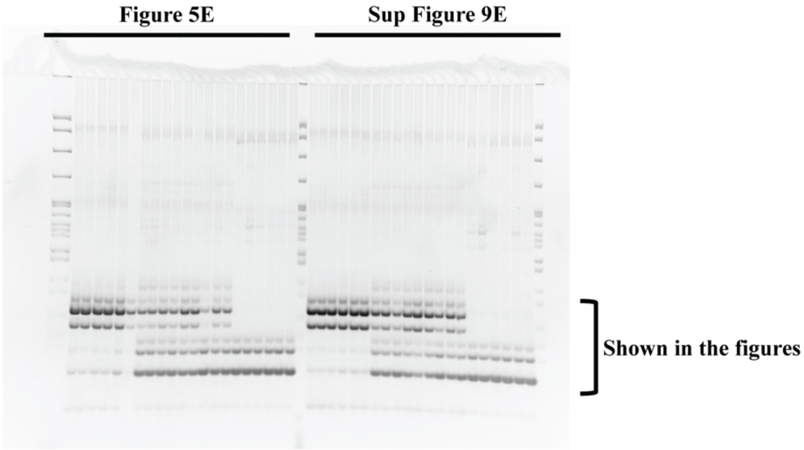

- Ryr2

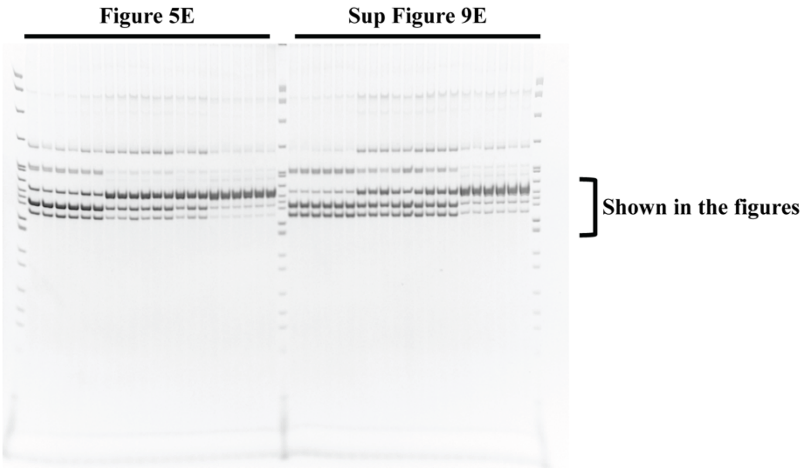

<u>Full unedited blots for Supplemental Figure 2C (left: with marker; right: image used in figure)</u>

- FLAG (ventricle; all samples were shown in the figure except the far left and far right ones)

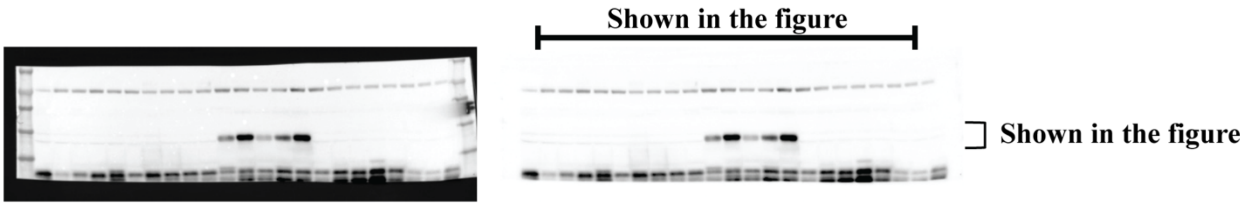

- MBNL1 (ventricle; all samples were shown in the figure except the far left and far right ones)

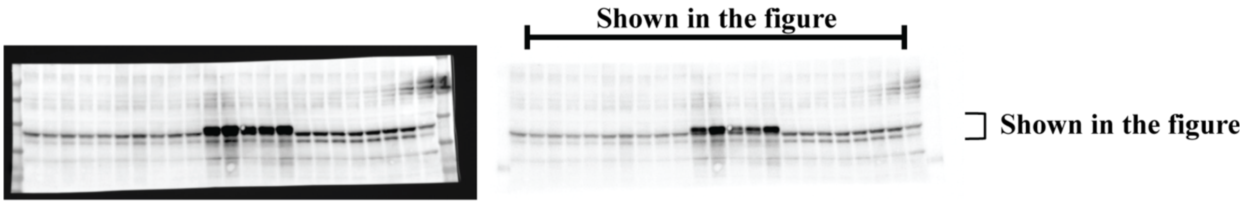

- MYC (ventricle; all samples were shown in the figure except the far left and far right ones)

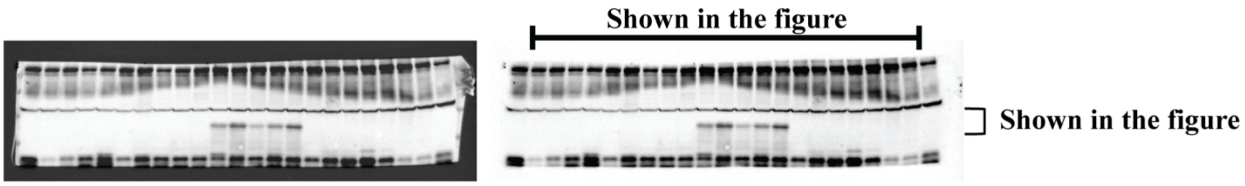

- MBNL2 (ventricle; all samples were shown in the figure except the far left and far right ones)

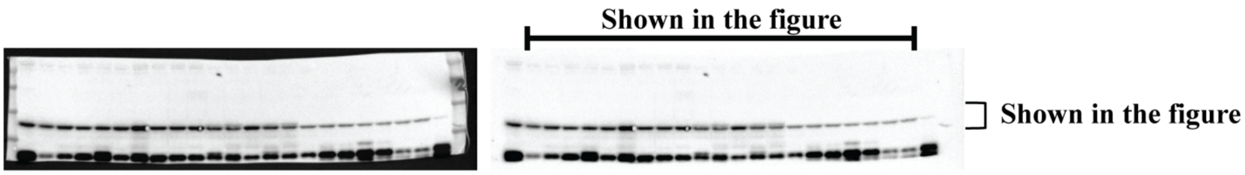

- mCherry (ventricle; all samples were shown in the figure except the far left and far right ones)

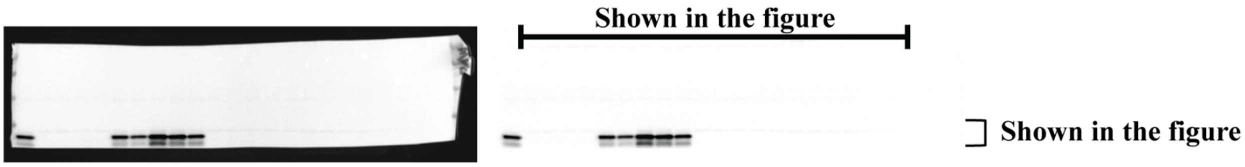

- Vinculin (ventricle; all samples were shown in the figure except the far left and far right ones)

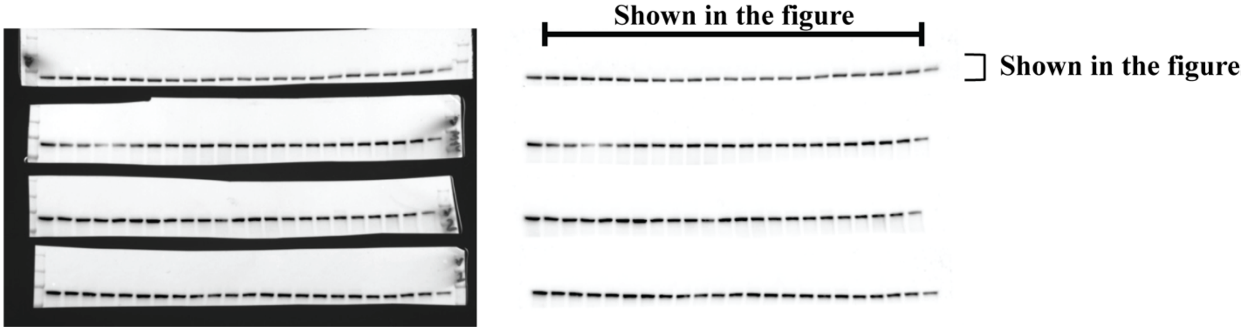

- FLAG (atria; all samples were shown in the figure except the first one on the left)

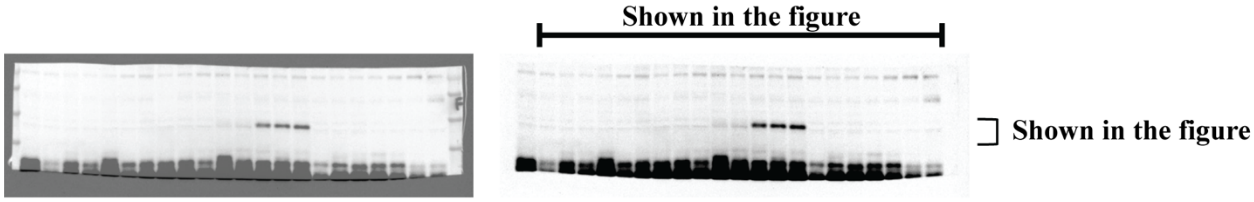

- MBNL1 (atria; all samples were shown in the figure except the first one on the left)

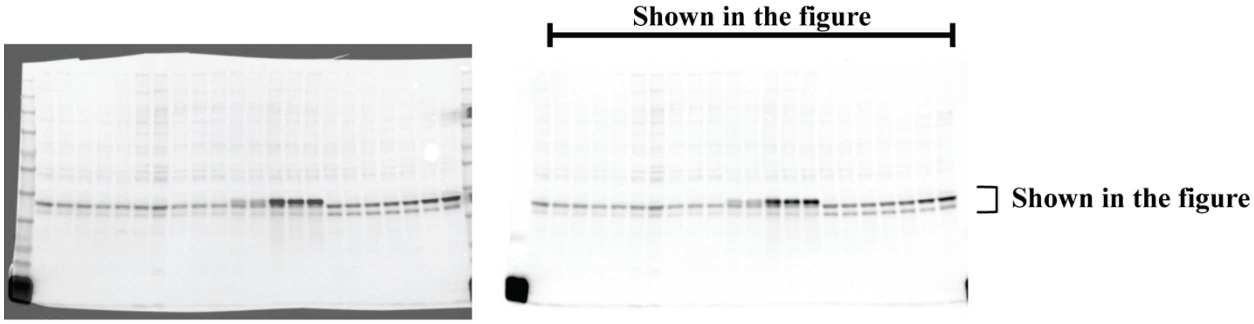

- MYC (atria; all samples were shown in the figure except the first one on the left)

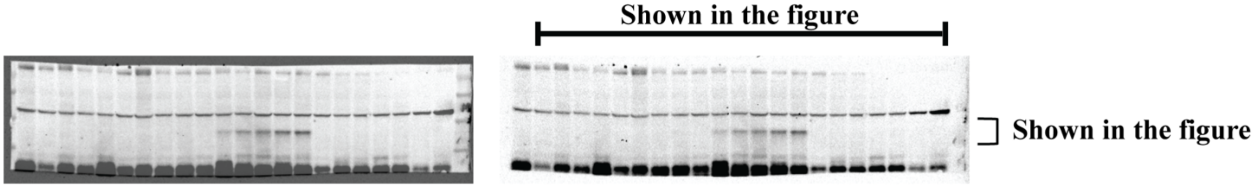

- MBNL2 (atria; all samples were shown in the figure except the first one on the left)

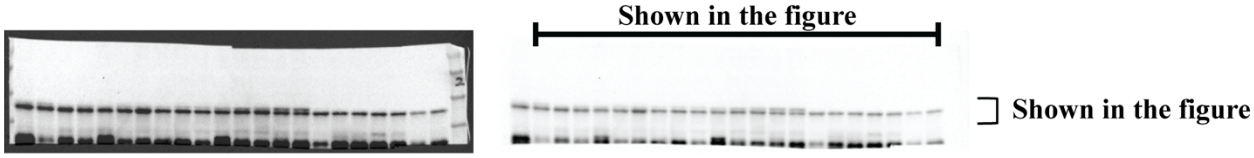

- mCherry (atria; all samples were shown in the figure except the first one on the left)

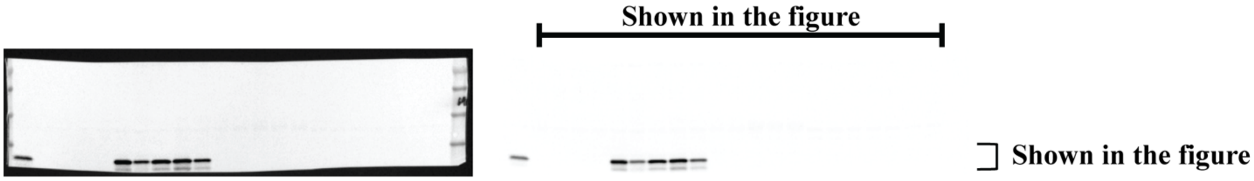

- Vinculin (atria; all samples were shown in the figure except the first one on the left)

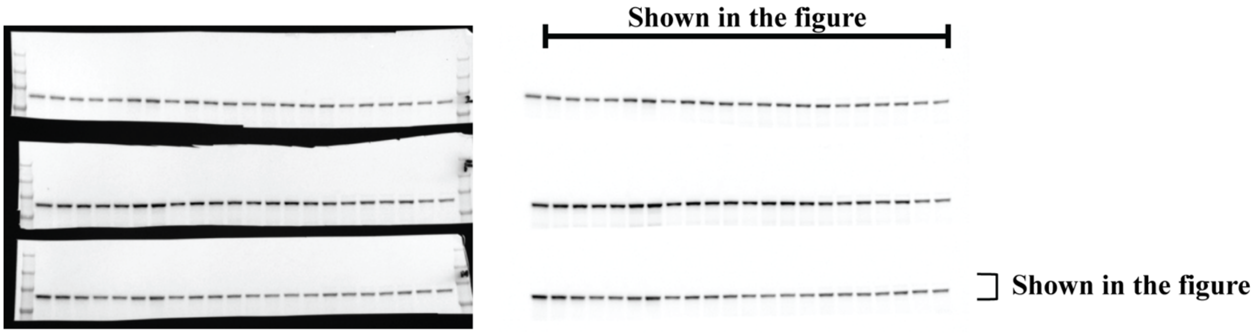

<u>**Full unedited gels for Supplemental Figure 10 and 11**</u>

- Kcnip2 E3

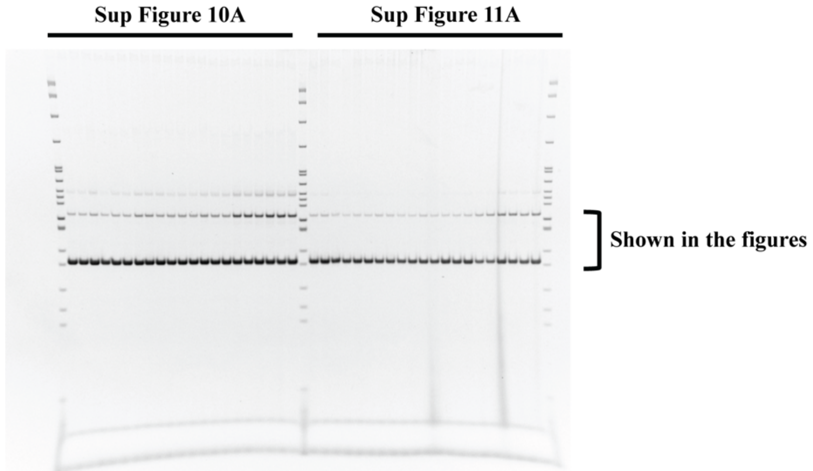

- Kcnd3 E6

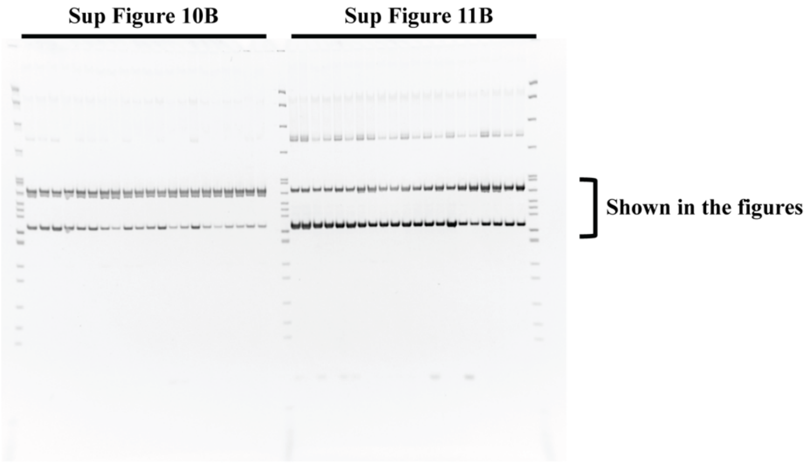

- Camk2d (-E14, 15, 16 vs +E15, 16)

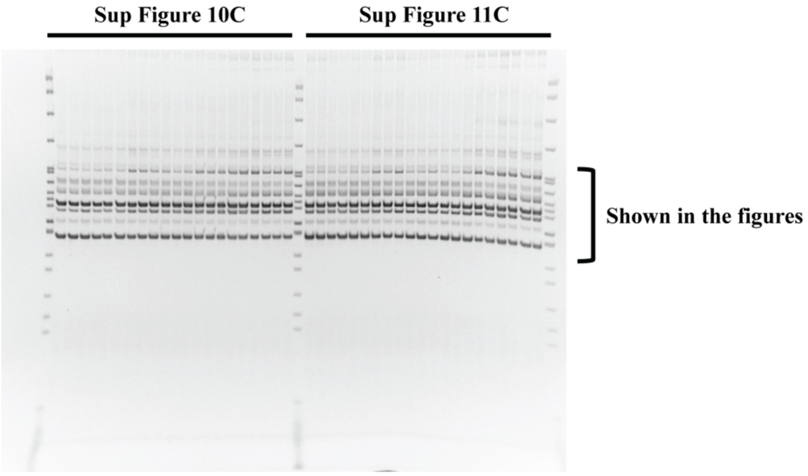

- Sorbs1 E25

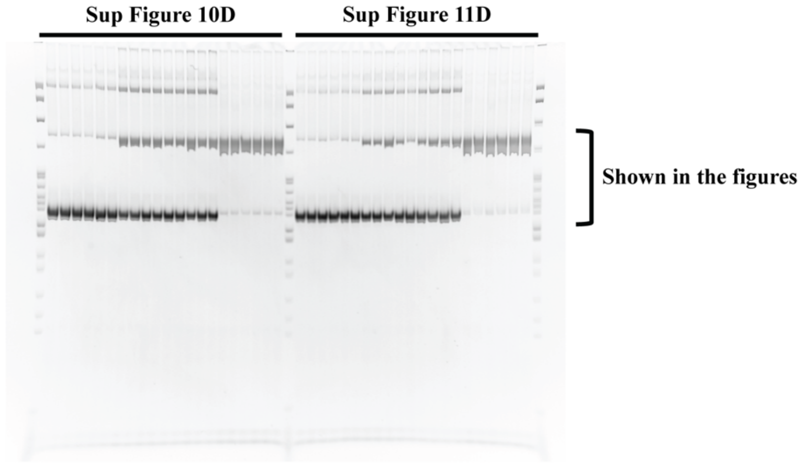

- Tmem63b E4 (ventricle; left) and Cacna1s E29 (ventricle; right)

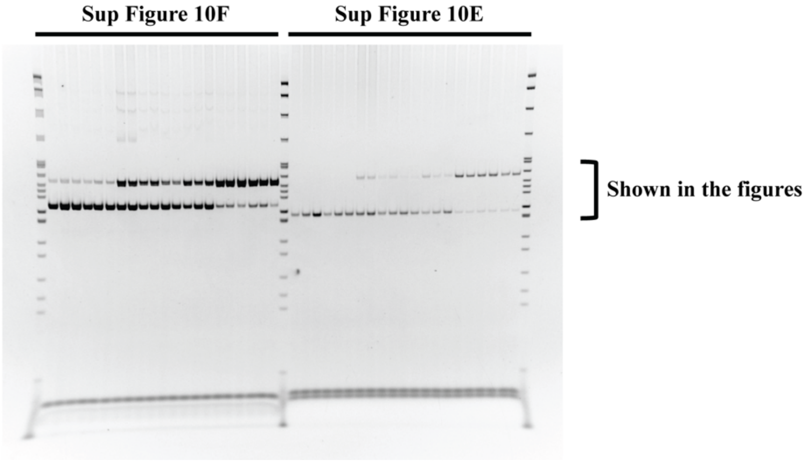

- Plekhn2 E7 (ventricle)

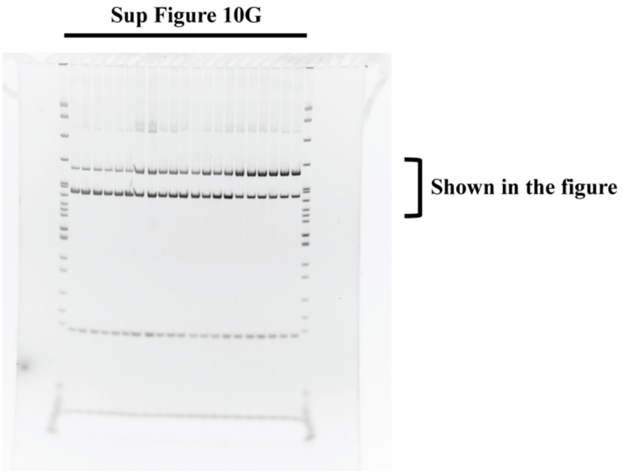

- Golga2 E3 (ventricle; left) and Dnm1l E17 (ventricle; right)

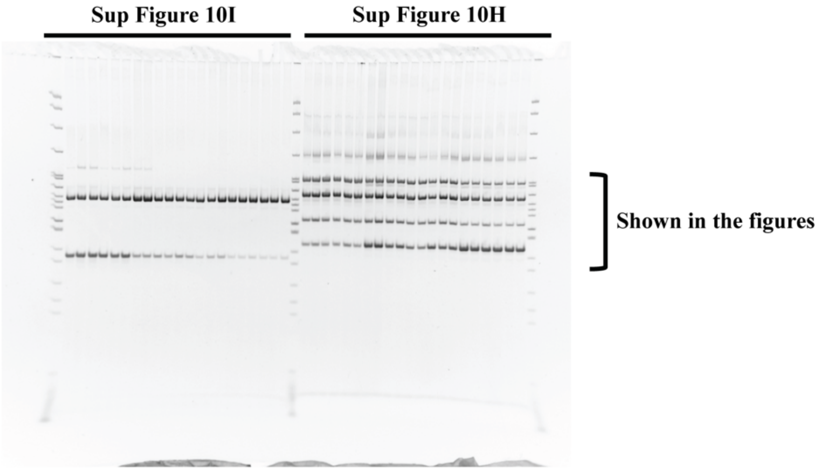

- Tmem63b E4 (atria; left) and Plekhm2 E7 (atria; right)

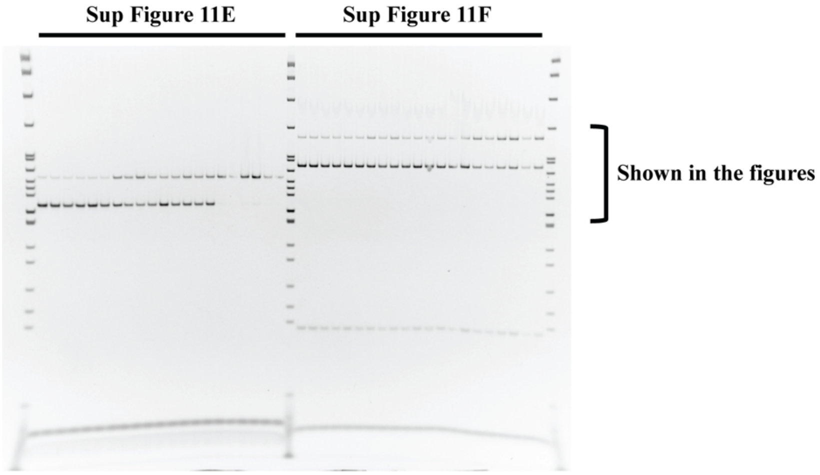

- Golga2 E3 (atria; left) and Dnm1l E17 (atria; right)

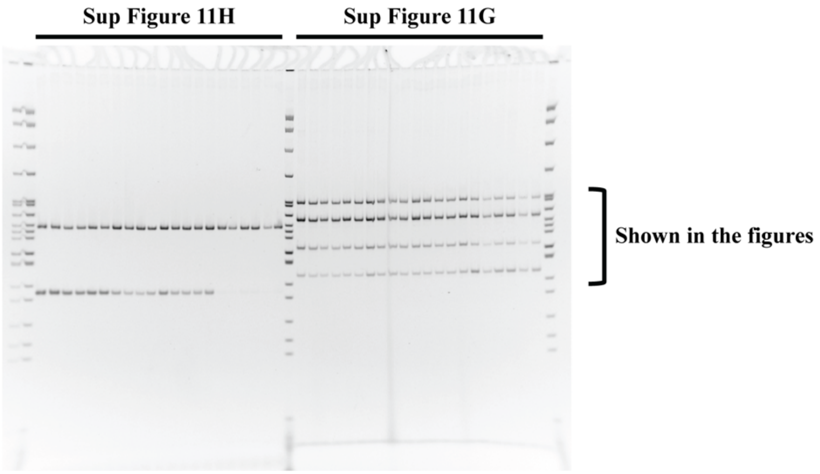

Supplement: Unedited blot and gel images [file jci-135-186416-s096.pdf]
